# Pre-synthetic redox control of structure and properties in copper TTFtt coordination polymers

**DOI:** 10.1039/d5sc03070f

**Published:** 2025-09-11

**Authors:** Ningxin Jiang, Saranya Velliyarat, Chen-Yu Lien, Ha L. Nguyen, Jan Hofmann, Jie-Hao Chen, Arun Ramanathan, Alexander S. Filatov, Henry S. La Pierre, Shrayesh Patel, Karena W. Chapman, Jan-Niklas Boyn, John S. Anderson

**Affiliations:** a Department of Chemistry, University of Chicago Chicago Illinois 60637 USA jsanderson@uchicago.edu; b Department of Chemistry, University of Minnesota Minneapolis Minnesota 55455 USA; c Department of Chemistry, Stony Brook University Stony Brook New York 11794 USA; d Pritzker School of Molecular Engineering, University of Chicago Chicago Illinois 60637 USA; e School of Chemistry and Biochemistry, Georgia Institute of Technology Atlanta Georgia 30332-0400 USA

## Abstract

Conductive coordination polymers (CPs) with sulfur-based ligands offer strong metal–ligand interactions and redox tunability, making them promising candidates for electronic applications. Tetrathiafulvalene-2,3,6,7-tetrathiolate (TTFtt) is a particularly attractive ligand. However, its strong metal–ligand covalency leads to rapid irreversible metal coordination, limiting control over structure and morphology. Here, we demonstrate structural control in Cu TTFtt CPs using a pre-synthetic redox control strategy. Two new copper-based CPs, CuTTFtt and Cu_2_TTFtt, have been synthesized and thoroughly characterized from differentially oxidized TTFtt synthons. CuTTFtt forms a 1D chain, while Cu_2_TTFtt adopts a 2D ribbon-like structure. Detailed spectroscopic studies confirm the structures of these materials as well as their ligand and metal oxidation states. Physical property measurements reveal that Cu_2_TTFtt exhibits higher conductivity than CuTTFtt. Furthermore, Cu_2_TTFtt also shows unusual diamagnetism which contrasts the paramagnetism observed in CuTTFtt and the related material NiTTFtt. Density functional theory (DFT) further elucidates the physical properties of these CPs and supports the observed conductivity trends. This study expands the structural landscape of TTFtt-based CPs and further establishes how redox-doping can tune CP structure and physical properties.

## Abstract 2

Conductive coordination polymers (CPs) with sulfur-based ligands offer strong metal–ligand interactions and redox tunability, making them promising candidates for electronic applications.

## Graphical abstract

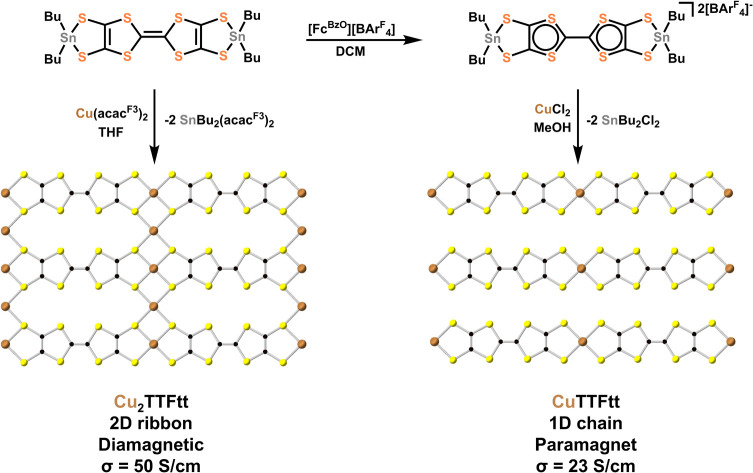

## Introduction

Conductive coordination polymers (CPs) are a promising class of materials for electronic and optoelectronic applications, including in sensing, energy storage, and electrocatalysis.^1–12^ Sulfur-based ligands are particularly attractive for constructing these materials due to their strong covalent bonding with transition metals, enabled by energetic matching and hence covalency between metals and sulfur.^13–19^ Many sulfur-containing ligands are also redox-active, which allows for further tuning of chemical and physical properties.^20–23^

Of many possible sulfur-rich ligands, tetrathiafulvalene-2,3,6,7-tetrathiolate (TTFtt), which combines a tetrathiafulvalene (TTF) core—a well-known motif in conductive molecules—with dithiolene coordination sites, is an excellent candidate for designing highly conductive materials.^23–25^ Several reports have investigated the combination of this linker with transition metals, but it typically exhibits rapid reaction with metal cations. This rapid irreversible reaction makes it difficult to control CP structure or morphology, and syntheses with TTFtt often yield amorphous black powders which can be difficult to characterize despite being highly conductive.^26–29^ Early synthetic efforts to generate TTFtt based CPs with both Ni and Cu resulted in conductive solids, but minimal insight into their electronic and geometric structure was obtained.^30^ This lack of insight is largely due to challenging structural characterization which can be particularly difficult with thiolate-based systems.^31–34^ Hoffmann and coworkers proposed several structures for TTFtt-based materials 40 years ago this year,^35^ but only a 1D chain structure of NiTTFtt has been experimentally demonstrated.^23^ Predictions of an alternative 2D sheet structure remain experimentally unverified.^36^

This dearth of detail presents a significant challenge in understanding (and controlling) the structure and properties of these materials. Dimensionality (1D, 2D, and 3D) plays a critical role over physical properties including both conductivity and magnetism, as demonstrated in both carbon-based materials and reticular structures.^37–39^ However, studies on the dimensionality of sulfur-based frameworks are rare. This difficulty in building structure–function relationships is made even more challenging as sulfur-based ligands often feature multiple accessible oxidation states which may change concurrently with changes in dimensionality.^20,21,40^ Many CP syntheses occur in aerobic conditions which can lead to *in situ* oxidation.^41–43^ This redox ambiguity complicates the determination of metal oxidation states, especially with redox-active metals such as Cu, where ambiguities in oxidation states are common in thiolate-based systems.^44,45^ The redox activity of ligands combined with the structural challenges mentioned above, make understanding and controlling the properties of TTFtt-based materials particularly challenging.

We recently employed a transmetalation and pre-synthetic doping strategy to successfully synthesize Ni CPs of TTFtt with variable TTFtt oxidation states.^40,46^ Using a pre-oxidized TTFtt transmetalating synthon provides NiTTFtt with a 1D chain structure where TTFtt is in a formally doubly oxidized state. While NiTTFtt displays high conductivity despite an amorphous structure, its reduced congener Li-NiTTFtt, with an overall TTFtt^4−^ ligand, displays intriguing photothermoelectric and thermoelectric properties.

This progress in understanding the structure and electronic properties of NiTTFtt motivates extending this synthetic control to other transition metal centers. Copper-thiolate CPs are known to exhibit electrical conductivity comparable to that of nickel-thiolate materials.^45,47,48^ We have therefore investigated copper coordination chemistry with TTFtt and synthesized two new materials, CuTTFtt and Cu_2_TTFtt. By employing similar pre-synthetic redox control of transmetalating TTFtt reagents, we can manipulate TTFtt oxidation states, with Cu_2_TTFtt containing TTFtt^3−^ linkers and CuTTFtt containing oxidized TTFtt^2−^ linkers. Thorough characterization, including X-ray absorption spectroscopy (XAS), X-ray photoelectron spectroscopy (XPS), and Raman spectroscopy, enable an accurate determination of ligand and copper oxidation states.

Structural analyses suggest that while CuTTFtt adopts a 1D chain structure similar to NiTTFtt, while Cu_2_TTFtt forms a 2D ribbon-like layered structure consistent with original structural models proposed by Hoffman and coworkers.^35^ Conductivity measurements demonstrate that Cu_2_TTFtt shows higher conductivity compared to CuTTFtt. In contrast to the dominant Pauli paramagnetism observed in NiTTFtt, Cu_2_TTFtt also shows diamagnetic behavior while CuTTFtt exhibits Curie–Weiss paramagnetism. Density functional theory (DFT) calculations were also employed to provide insight into the different physical properties of NiTTFtt, CuTTFtt, and Cu_2_TTFtt and validate the observed experimental trends.

These findings validate and expand the known structural types for TTFtt-based CPs and also elucidate how these structures influence charge transport properties. Moreover, the different morphologies observed for these copper-based CPs suggest that linker redox-tuning is an important strategy for controlling structure. This study motivates continued investigations into how the structure and metal identity of TTFtt-based materials dictates magnetic coupling and novel emergent properties at the interface of conductivity and magnetism.

## Results and discussion

### Synthesis of CuTTFtt and Cu_2_TTFtt

**Fig. 1:**
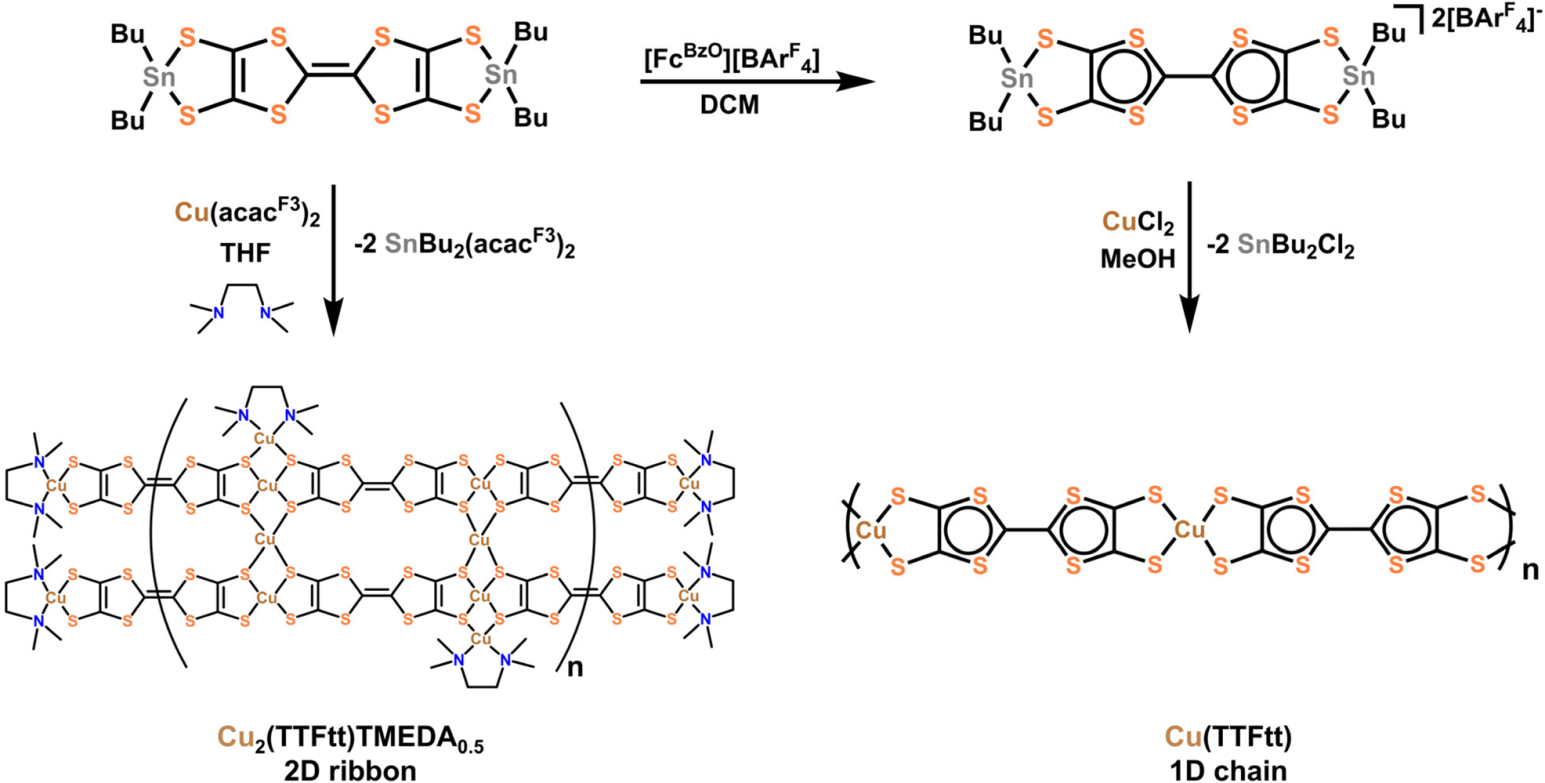
Synthetic scheme for Cu_2_TTFtt (left) and CuTTFtt (right).

The syntheses of CuTTFtt and Cu_2_TTFtt utilize transmetalation with TTFtt(SnBu_2_)_2_^*n*+^ reagents (*n* = 0 or 2) due to the ability to program a desired TTFtt redox state prior to CP synthesis (Fig. 1).^22^CuTTFtt was synthesized following a similar procedure to NiTTFtt.^23^ First, TTFtt(SnBu_2_)_2_ was oxidized using Fc^Bzo^BAr^F^_4_ (Fc^Bzo^ = benzoylferrocenium and BAr^F^_4_ = tetrakis[3,5-bis(trifluoromethyl)phenyl]borate) in dichloromethane (DCM). Separately, CuCl_2_ was dissolved in methanol (MeOH) and subsequently mixed with a DCM solution of oxidized TTFtt(SnBu_2_)_2_. A black powder (CuTTFtt) quickly formed and was isolated after workup.

For Cu_2_TTFtt, two equivalents of Cu(acac^F3^)_2_ (acac^F3^ = trifluoroacetylacetonate) were mixed with excess tetramethylethylenediamine (TMEDA) in tetrahydrofuran (THF), then combined with one equivalent of TTFtt(SnBu_2_)_2_ in THF to immediately generate a dark powder. It is worth noting that the addition of TMEDA to a Cu(acac^F3^)_2_ solution initially results in an immediate color change from blue to green, suggesting the formation of [(Cu(TMEDA)_2_)]^2+^. The isolated dark green (nearly black) powder was dried at 70 °C to yield Cu_2_TTFtt.

### Composition

X-ray fluorescence (XRF) analysis was initially used to determine the elemental composition of Cu_2_TTFtt and CuTTFtt (SI, Fig. S1 and Table S1). The Cu : S ratio is 1 : 3.78 for Cu_2_TTFtt and 1 : 9.7 for CuTTFtt corresponding to Cu : TTFtt ratios of ∼2 : 1 and ∼1 : 1, respectively. Minimal Sn content (<1% atomic ratio relative to Cu) was detected in both samples, indicating that copper effectively transmetalates tin in the TTFtt linkers. Combustion analysis for CuTTFtt reveals 19.99(8)% carbon, and 0.49(2)% hydrogen, suggesting negligible organic components beyond TTFtt. These results align with minimal mass loss observed below 200 °C in thermogravimetric analysis (TGA, Fig. S2). Based on this data, the chemical formula of CuTTFtt is most consistently assigned as CuC_6_S_8_ (Cu(TTFtt)).

For Cu_2_TTFtt, combustion analysis yields 21.36(1)% carbon, 2.72(3)% nitrogen, and 1.75(3)% hydrogen. These results suggest some additional organic component beyond a limiting formula of Cu_2_C_6_S_8_. Combined with a ∼10% mass loss observed at ∼200 °C in TGA (Fig. S2), we propose a chemical formula for Cu_2_TTFtt as Cu_2_C_6_S_8_(C_6_H_16_N_2_)_0.5_ (Cu_2_(TTFtt)(TMEDA)_0.5_) with the inclusion of 0.5 TMEDA molecules per formula unit. The inclusion of TMEDA suggests a fundamentally different structure for Cu_2_TTFtt. The proposed chemical formulas of both CuTTFtt and Cu_2_TTFtt match well with the combustion analysis results shown in Table S2.

### Structural analysis

**Fig. 2:**
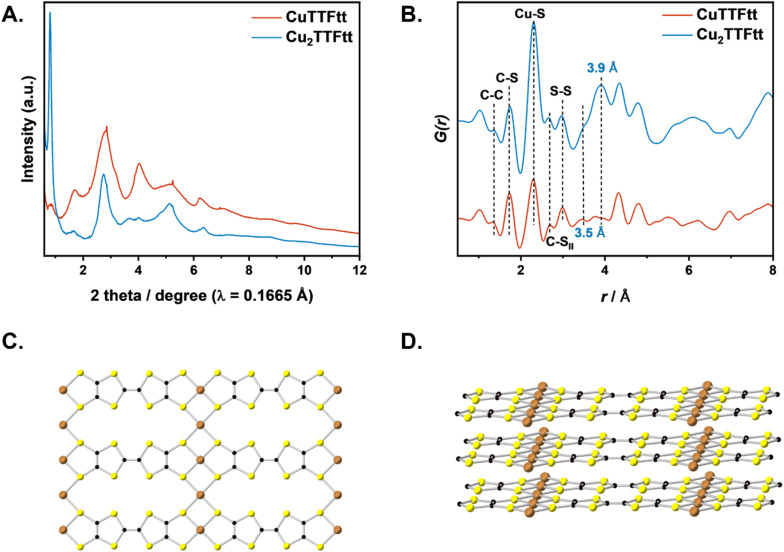
(A) Synchrotron (*λ* = 0.167 Å) PXRD patterns and of Cu_2_TTFtt and CuTTFtt, (B) PDF of Cu_2_TTFtt and CuTTFtt. Structural model of Cu_2_TTFtt viewed along *c*-axis (C) and viewed from *a*-axis (D).

Both lab-based (Cu source, *λ* = 1.541 Å) and synchrotron (*λ* = 0.167 Å) sources were used for powder X-ray diffraction (PXRD) measurements to elucidate the structures of CuTTFtt and Cu_2_TTFtt. Lab-based PXRD analysis of CuTTFtt reveals no sharp Bragg peaks with only a very broad feature between about 23 and 30° 2*θ* that is indicative of a structure with low crystallinity (Fig. S3A). In contrast, Cu_2_TTFtt reveals two broad peaks centered at about 7.6° and 25.6°, suggesting a somewhat higher degree of crystallinity compared to CuTTFtt and most reported TTFtt-based CPs. The synchrotron PXRD patterns reveal substantially more diffraction peaks (Fig. 2A). The presence of TMEDA was found to be essential for obtaining crystalline samples (Fig. S3B). The PXRD pattern and carbon content of Cu_2_TTFtt remain unchanged after heating at 160 °C for 6 hours despite the boiling point of TMEDA being approximately 120 °C. This suggests that TMEDA strongly binds to the framework, playing a critical structural role in Cu_2_TTFtt.

Pair distribution function (PDF) data (Fig. 2C and S5A) reveal local range order in these CPs. The low r region of the PDF data for both materials contains features at ∼1.4 Å, 1.8 Å, 2.3 Å, 2.7 Å and 3.0 Å that correspond to the C–C, C–S, Cu–S, C⋯S, and S⋯S distances, respectively (Fig. 2D and S5). These peaks correspond to intra-chain atomic distances within CuTTFtt and Cu_2_TTFtt and thus verify the presence of 1D chains in both materials. The PDF of CuTTFtt can be well-described by a single-chain model derived from NiTTFtt (Fig. S6). The difference in peak position between model and data around 2.3 Å is due to different Cu–S bond lengths compared with Ni–S distances from the NiTTFtt model.^23^

**Fig. 3:**
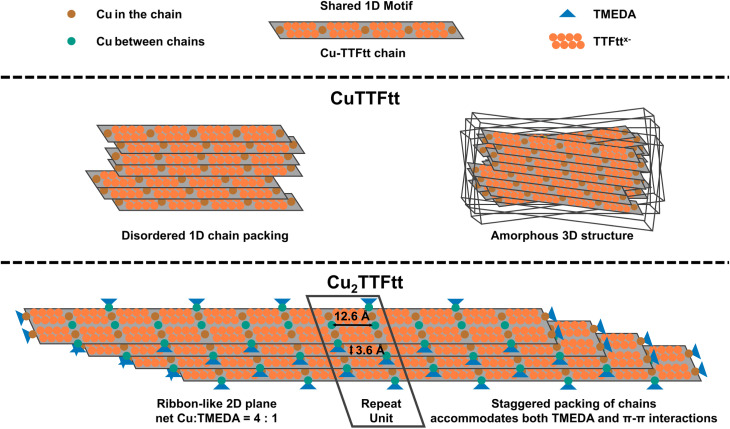
Demonstration of proposed structural models of CuTTFtt (middle) and Cu_2_TTFtt (bottom) based on PXRD, PDF and elemental analysis.

The PDF of Cu_2_TTFtt shows distinct differences in local order from the 1D materials, particularly in the intensity of the Cu–S peak at 2.3 Å and the presence of additional peaks at 3.5 Å and 3.9 Å. A similar 3.9 Å distance has been associated with a side-by-side ligand arrangement in other TTF based materials,^49^ and so the presence of this feature in Cu_2_TTFtt suggests the presence of such a side-by-side TTFtt arrangement. Combined with the increased Cu–S intensity observed in the PDF of Cu_2_TTFtt, we propose that these increased peak intensities correspond to additional Cu^2+^ ions that bind to sulfur in between negatively charged CuTTFtt^2−^ chains in a side-by-side arrangement (Fig. 2C, D and 3). As previously mentioned, a structural model for tetrathiolate-based materials with a metal-to-ligand ratio of 2 : 1 was previously proposed by Hoffmann and coworkers in 1985, but experimental validations have been lacking until the present example for Cu_2_TTFtt. This model has also been proposed as a potential structure for [Cu_*x*_(Cu-ETT)] systems.^35,36^ We therefore propose a related 2D model for Cu_2_TTFtt with additional Cu–S bonds, which is consistent with the larger peak at 2.3 Å in the PDF data (Fig. S5B). This model consists of a 2D layered framework where copper cations connect 1D chains (Fig. 2C, D and S7). Consistent with prior data on TTFtt materials, the interlayer distance is 3.62 Å and the distance between neighboring Cu centers is 12.6 Å within a Cu–TTFtt chain. It should be noted that this construct is an idealized highly symmetric model of the material and requires a perfect 1D chain length match and alignment when propagating along the second dimension. Any mismatch in these distances/alignments would lead to disorder in the material and formation of amorphous to semi-crystalline materials as observed experimentally.

The simulation of the PXRD pattern based on this idealized model using *Pmmm* space group and a unit cell of *a* = 6.67 Å, *b* = 12.61 Å, *c* = 3.62 Å (*V* = 304.3 Å^3^) reasonably reproduces the general features of the experimental diffraction pattern. This motivated the use of this model for a crude Rietveld refinement. This analysis is admittedly limited due to the poor crystallinity of Cu_2_TTFtt, but the Rietveld refinement does show a reasonable fit with the experimental data which provides some validation of the proposed structural model (Fig. S9). The refined unit cell parameters are *a* = 6.613(11) Å, *b* = 12.991(8) Å, *c* = 3.6136(20) Å (*V* = 310.4(6) Å^3^) with *R* and *wR* factors of 0.042 and 0.051 respectively. The only sharp Bragg peak is located at *Q* = 0.53 Å^−1^ and is assigned to a (010) reflection suggesting a more precise long-order arrangement of 1D chains in the structure compared to all other dimensions. The crystallite size (as refined over the whole powder pattern) is ∼7 nm, which is indicative of 5 to 6 Cu–TTFtt motifs in a single chain or ∼20 Cu_2_TTFtt layers. While this domain size is reasonable for a material with defects and disorder, it should be emphasized that this value is highly sensitive to both the sample's low crystallinity and to the limitations of our structural model. As such, the reported crystallite size should be regarded as an approximate lower bound rather than an absolute measurement.

While these X-ray analyses provide a reasonable structure for Cu_2_TTFtt, composition studies reveal a significant amount of TMEDA which is unaccounted for in the structural model. Attempts to incorporate TMEDA into the 2D plane or between the 2D planes do not produce physically reasonable models due to steric clashes. We instead propose that TMEDA binds exclusively to copper at the edge sites of Cu_2_TTFtt. TMEDA is known to act as a bidentate ligand for copper, forming complexes such as [Cu(TMEDA)L_*x*_] or [Cu(TMEDA)_2_]^1+/2+^. When L represents sulfur-based ligands, a square planar geometry is reasonably expected in [Cu(TMEDA)L_2_].^50^ Given that Cu cations bind strongly to sulfur-based ligands, we propose that Cu(TMEDA)^1+/2+^ resides at the terminal positions of each Cu–TTFtt chain in Cu_2_TTFtt. A structural model was generated to allow AA stacking of Cu–TTFtt chains with TMEDA termination, as shown in Fig. S10. After structural optimization, the interlayer distance increased to 6.5 Å, which is longer than the expected 3.6 Å from PXRD data and suggests that Cu(TMEDA) complexes cannot stack directly on top of each other from different chains (Fig. S10). However, a structural model with TMEDA present only at terminal positions cannot account for the Cu : TMEDA ratio of 4 unless each chain consists of only four TTFtt anions, which is inconsistent with the strong (010) peak observed at 0.80° 2*θ* (*l* = 0.167 Å). The experimentally observed Cu : TMEDA ratio can only be achieved if two Cu–TTFtt chains are coupled in Cu_2_TTFtt, as illustrated in Fig. 3 and S12. The resulting structural model, shown in Fig. 3, demonstrates a chemical formula of Cu_2_TTFtt(TMEDA)_0.5_, which aligns well with the compositional analysis and avoids TMEDA steric clashes. Thus, the combined experimental data support that Cu_2_TTFtt adopts a 2D ribbon-like layered structure similar to that shown in Fig. 3.

We note that, despite the stoichiometric amount of TMEDA present, no distinct peak corresponding to TMEDA can be identified in the PDF analysis of Cu_2_TTFtt (Fig. 2B). This absence can be attributed to two factors. Firstly, reasonable Cu–N bond lengths (∼1.9 Å) have significant overlap with the numerous C–S bonds in the material. Secondly, the number of proposed Cu–N bonds is much smaller than the other bonds represented in the PDF analysis, resulting in a lower signal intensity that cannot be directly observed.

To further characterize the structures of Cu_2_TTFtt and CuTTFtt, we carried out Cu K-edge XAS measurements and analyzed the EXAFS data (Fig. S13, S14, Tables S3 and S4). For CuTTFtt, the best-fit results yield a Cu–S distance of 2.28 Å, a Cu–C distance of 3.13 Å, and an average Cu coordination number of 4.0 ± 0.2. These values are consistent with previously reported Cu–S bond lengths and with our PDF analysis,^51^ confirming that Cu in CuTTFtt adopts a square-planar coordination environment. This finding further supports the conclusion that the structure of CuTTFtt closely resembles that of NiTTFtt, forming a one-dimensional chain-like arrangement.

For Cu_2_TTFtt, the EXAFS fitting gives a Cu–N distance of 1.76 Å, a Cu–S distance of 2.27 Å, a Cu–Cu distance of 2.95 Å, and a Cu–C distance of 3.14 Å, with an average Cu coordination number of 4.1 ± 0.4. The Cu–S bond length again agrees well with the PDF analysis, confirming that Cu also adopts a square-planar geometry in this compound. The relative ratio of Cu–N to Cu–S bonds (1 : 8) is close to the theoretical value of 1 : 7, consistent with the presence of stoichiometric TMEDA in the structure. Together, these EXAFS results provide direct structural support that Cu_2_TTFtt adopts a two-dimensional ribbon-like framework.

### Ligand redox state analysis

One advantage of using TTFtt(SnBu_2_)_2_^*n*+^ transmetalating agents is the well-defined TTF oxidation state which should translate into the resulting CP. However, *in situ* redox chemistry can frequently occur, and so rigorous characterization is necessary to confirm the proposed oxidation states of the TTFtt linker and Cu centers in the obtained materials. Normally, such an analysis would begin with characterization of the Cu oxidation states. However, the oxidation states of Cu centers can be notoriously difficult to concretely assign, particularly in covalent sulfur-based materials. Indeed, both XPS and XAS data provide somewhat ambiguous results (see SI Section 4).^44,45,52,53^

**Fig. 4:**
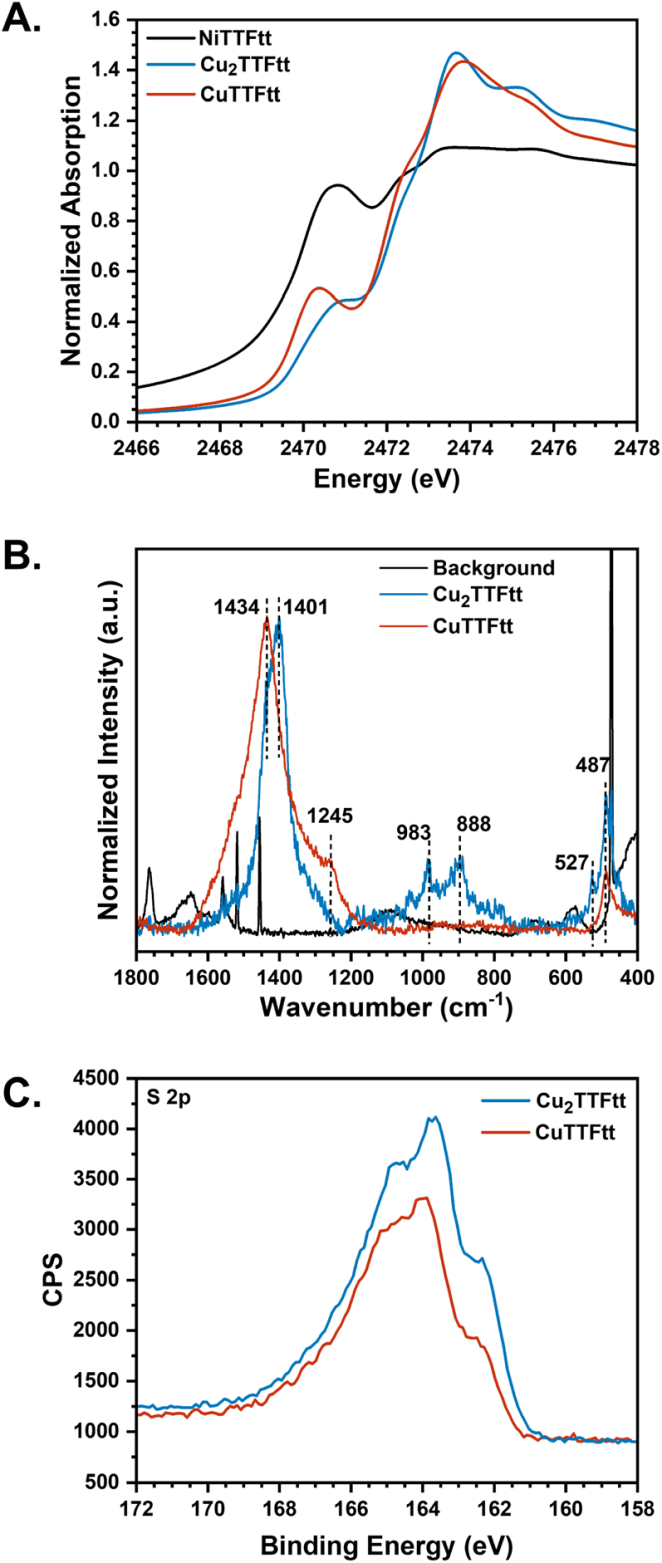
Spectroscopic studies TTFtt ligand redox states. (A) Sulfur K-edge XAS, (B) Raman spectra, and (C) S 2p XPS spectra of Cu_2_TTFtt and CuTTFtt.

Recent sulfur K-edge XAS studies on Ni-TTFtt molecules indicate that the first pre-edge feature in doubly oxidized TTFtt^2−^ appears ∼0.6 eV lower in energy than the pre-edge feature in both neutral TTFtt^4−^ and singly oxidized TTFtt^3−^.^54^ This provides a useful benchmark to examine the oxidation state of TTFtt linkers in these copper-based materials. We therefore collected sulfur K-edge XAS data on both Cu_2_TTFtt and CuTTFtt (Fig. 4A). Molecular analogs of copper TTFtt compounds have not yet been successfully synthesized, so we compared the observed spectroscopic features to those of analogous nickel compounds for interpretation. For CuTTFtt, the first pre-edge feature is observed at 2470.4 eV, while in Cu_2_TTFtt, a shoulder-like feature appears at approximately 2471.2 eV. The 0.8 eV energy difference between the two samples strongly suggests that the TTFtt motifs are in different overall redox states. By comparison, the sulfur K-edge pre-edge feature for NiTTFtt appears at 2470.7 eV, supporting that the linkers in CuTTFtt are best assigned with a formal oxidation state of TTFtt^2−^. However, the pre-edge positions for TTFtt^3−^ and TTFtt^4−^ are similar, and so the redox state of TTFtt in Cu_2_TTFtt cannot be determined from sulfur K-edge XAS data alone.

To further investigate the formal TTFtt redox state, Raman spectroscopy and XPS were employed. Infrared signals were difficult to interpret due to broadening, presumably from high reflectivity/shielding from potential metallic character (Fig. S15). The Raman spectra for Cu_2_TTFtt and CuTTFtt are more informative and are shown in Fig. 4B. Both compounds show peaks between 1400-1450 cm^−1^ which can be assigned to C–C vibrations.^55^ For Cu_2_TTFtt, this feature is centered at 1401 cm^−1^, compared to 1434 cm^−1^ in CuTTFtt. This shift to a lower wavenumber in Cu_2_TTFtt suggests differences in C–C bonding order, likely indicating longer C–C bond lengths in Cu_2_TTFtt and thus a more reduced formal oxidation state of the TTFtt linkers. Similar trends have been observed in NiTTFtt coordination complexes, where oxidation of the TTFtt motif decreases C–C bond length.^40^ In CPs such as NiTTFtt (TTFtt^2−^) and Li-NiTTFtt (TTFtt^4−^), oxidized TTFtt CPs consistently show C–C vibrations at higher wavenumbers.^23,40^ Thus, the higher frequency features observed in CuTTFtt*vs.*Cu_2_TTFtt further support more reduced TTFtt linkers in Cu_2_TTFtt.

Additional peaks are present around 900 cm^−1^ which can be assigned to C–S vibrations. These features are present in Cu_2_TTFtt and Li-NiTTFtt but are absent in CuTTFtt,^55^ and so we hypothesize that they may serve as a characteristic Raman signal for reduced TTFtt linkers (*i.e.* TTFtt^4−^).^40^ Overall, the observed Raman features strongly support the assignment of TTFtt in Cu_2_TTFtt as having a lower redox state than in CuTTFtt. Both samples also show a peak at 487 cm^−1^ which arises from Cu–S vibrations. However, Cu_2_TTFtt shows an extra peak at 527 cm^−1^ which indicates an additional sulfur ligation environment in Cu_2_TTFtt and further supports the proposed 2D ribbon structure.

Sulfur 2p XPS data was finally collected to further corroborate the TTFtt oxidation states (Fig. 4C). Notably, both Cu_2_TTFtt and CuTTFtt exhibit a main peak accompanied by two shoulder-like features. Deconvolution of the spectra into three distinct sulfur chemical states yields a reasonable fit, as shown in Fig. S17. Based on previous XPS analyses of poly[Cu_*x*_(Cu-ETT)],^36^ the first two sets of doublets can be assigned to the reduced terminal sulfur (162.5 eV) and the oxidized terminal sulfur (164.1 eV) in the TTFtt ligand. The broad doublets at higher binding energy (164.8 eV) correspond to sulfur within the TTF core. Notably, the ratio of oxidized sulfur to reduced sulfur, determined from peak areas, increases from 1.20 in Cu_2_TTFtt to 2.15 in CuTTFtt, confirming a more oxidized redox state of TTFtt in CuTTFtt. Interestingly, the 162 eV feature, which intensifies with increasing oxidation of TTFtt, has also been observed in NiTTFtt (Fig. S18), poly[Cu_*x*_(Cu-ETT)], and Li_*x*_Fe_3_(THT)_2_.^21,23,36^ This observation highlights XPS as a powerful tool for studying ligand redox states.

Finally, we examined the Cu K-edge XANES spectra of CuTTFtt and Cu_2_TTFtt (Fig. S20). The absorption edge of Cu_2_TTFtt is clearly shifted to lower energy relative to that of CuTTFtt. Since the Cu K-edge position decreases with decreasing oxidation state, this shift indicates the presence of a more reduced copper component in Cu_2_TTFtt. Together with the spectroscopic evidence for TTFtt^2−^ ligands in CuTTFtt, this strongly supports a Cu^2+^ assignment in this compound, giving an overall formal redox state of (Cu^2+^)(TTFtt^2−^). For Cu_2_TTFtt, the ligand is expected to be either TTFtt^3−^ or TTFtt^4−^; considering the lower edge position and the presence of a more reduced copper species, the most reasonable assignment is a mixed-valent state of (Cu^2+^)(Cu^+^)(TTFtt^3−^).^51^

### Physical property studies

**Fig. 5:**
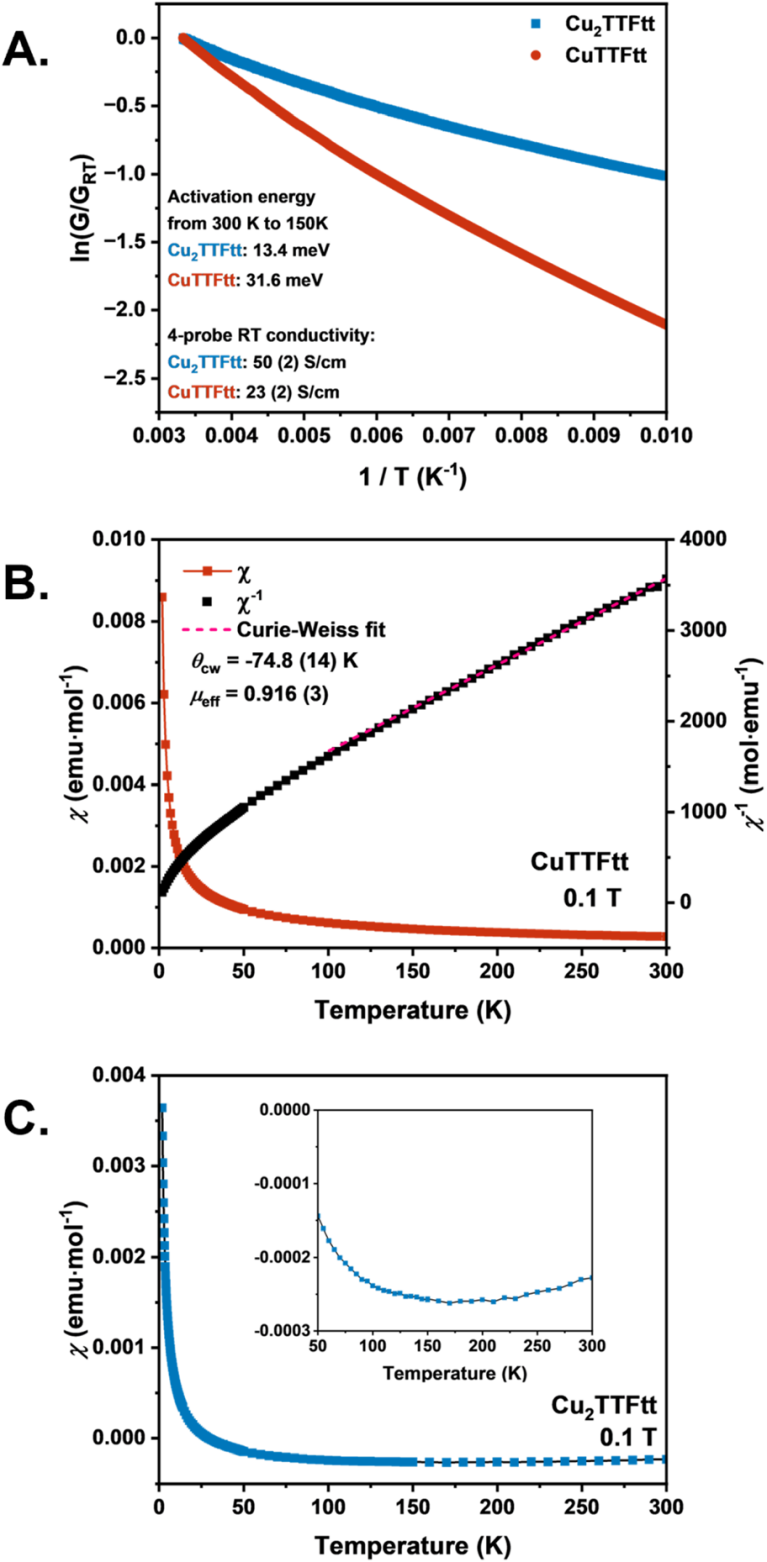
Charge transport and magnetic properties of CuTTFtt and Cu_2_TTFtt. (A) Variable temperature resistance measurements. (B) Variable temperature magnetic susceptibility measurements of CuTTFtt at 0.1*T*. Curie–Weiss fit in pink. (C) Variable temperature magnetic susceptibility measurements of Cu_2_TTFtt at 0.1*T*. Inset shows negative susceptibility, supporting diamagnetism.

The electrical and magnetic properties of Cu_2_TTFtt and CuTTFtt were then analyzed (Fig. 5). A room-temperature four-point electrical conductivity of 50(2) S cm^−1^ for Cu_2_TTFtt and 23(2) S cm^−1^ for CuTTFtt were obtained. These values are high among those reported for most conductive CPs. Comparison of these values to those for NiTTFtt materials is informative. The reduced compound Li-NiTTFtt, which has TTFtt in a reduced state similar to Cu_2_TTFtt, shows a considerably lower conductivity (10(1) S cm^−1^), while NiTTFtt with oxidized TTFtt linkers is dramatically more conductive (4.7(3) × 10^2^ S cm^−1^).^23,40^ Since Li-NiTTFtt adopts a 1D chain structure, the higher conductivity of Cu_2_TTFtt compared to both Li-NiTTFtt and CuTTFtt suggests that the 2D ribbon-like structure enhances charge transport.

Additional data were then collected to further understand the charge transport properties of CuTTFtt and Cu_2_TTFtt. Both materials show increased resistance with decreasing temperature (Fig. 5A). Fitting the temperature-dependent conductivity data from 300 K to 150 K yields activation energies of 13.4 meV for Cu_2_TTFtt and 31.6 meV for CuTTFtt. The smaller activation energy in Cu_2_TTFtt suggests either a higher carrier density or mobility in Cu_2_TTFtt than in CuTTFtt. The UV-vis-NIR spectra (Fig. S21) show no sharp absorbance drop for either compound, indicating a band gap smaller than 0.62 eV, consistent with the small activation energy values. Ultraviolet photoelectron spectroscopy (UPS) allows for the determination of work functions of 4.27 eV for Cu_2_TTFtt and 4.47 eV for CuTTFtt (Fig. S22). Notably, for both compounds, the counts per second (CPS) drop to background levels at 0 eV, indicating a low or zero density of states at the Fermi level. The valence band maxima were determined to be 0.23 eV for Cu_2_TTFtt and 0.63 eV for CuTTFtt. These results suggest that both compounds are small-bandgap semiconductors, differing from the glassy metallic behavior observed in NiTTFtt. This is also consistent with the lower electrical conductivity values observed for both compounds compared to NiTTFtt. The Seebeck coefficients are 10.9(2) μV K^−1^ for Cu_2_TTFtt and −2.5(5) μV K^−1^ for CuTTFtt. In comparison, Li-NiTTFtt exhibits a Seebeck coefficient of 10 μV K^−1^, while NiTTFtt has a value of −3.6 μV K^−1^. Notably, materials where TTFtt is in a more reduced state (Cu_2_TTFtt and Li-NiTTFtt) exhibit p-type behavior, whereas those where TTFtt is in a more oxidized state (CuTTFtt and NiTTFtt) exhibit n-type behavior. This trend underscores the critical role of ligand redox states in tuning the electronic structure and determining the charge-carrier type in TTFtt-containing materials.

The magnetic properties of both CuTTFtt and Cu_2_TTFtt were also investigated, as shown in Fig. 5B and C. In contrast to NiTTFtt, which contains square planar Ni^2+^ cations in a closed shell diamagnetic 3d^8^ electron configuration, both copper compounds presented here contain Cu^2+^ cations, which have a 3d^9^ electronic configuration and are *S* = 1/2, potentially leading to magnetic behavior. For CuTTFtt the temperature dependence of the magnetic susceptibility (*χ*) increases monotonically with decreasing temperature from 300 to 1.8 K, consistent with paramagnetic behavior (Fig. 5B). As CuTTFtt exhibits high electrical conductivity, a small band gap, and *S* = 1/2 spin centers, some combination of both Pauli and Curie–Weiss paramagnetism is reasonable.^56^ Fitting the data from 150 K to 300 K using the Curie–Weiss law yields a Curie–Weiss temperature (*θ*_CW_) of −74.8(14) K, Curie constant of 0.105 emu K mol^−1^ and an effective magnetic moment of 0.916(3)*μ*_B_/Cu^2+^, which is lower than the expected 1.73*μ*_B_/Cu^2+^ for *S* = 1/2 spins. The *χT* value at room temperature is 0.08 emu K mol^−1^ (Fig. S25C), which is also significantly lower than the expected spin-only value of 0.375 emu K mol^−1^. The field dependence of magnetization at 1.8 K slowly increases nonlinearly to 0.037*μ*_B_/Cu^2+^ up to 7*T* (Fig. S25D).

To differentiate the contributions of Pauli and Curie–Weiss paramagnetism in TTFtt^2−^-based CPs, a modified Curie–Weiss law, *χ* = *C*/(*T* − *θ*_CW_) + *χ*_0_, incorporating a temperature-independent component (*χ*_0_), was used to fit the temperature dependence of the magnetic susceptibility for both CuTTFtt and NiTTFtt (Fig. S26) from 100 K to 300 K.^56^ Diamagnetic corrections were applied prior to fitting, ensuring that *χ*_0_ primarily represents the Pauli paramagnetic contribution. For NiTTFtt, *C* = 0.0797 emu K mol^−1^, *χ*_0_ = 3.28 × 10^−4^ emu mol^−1^ and *θ*_CW_ = 3.6 K, indicating paramagnetism dominated by Pauli contributions. In contrast, for CuTTFtt, *C* = 0.0739 emu K mol^−1^, *χ*_0_ = 3.40 × 10^−5^ emu mol^−1^ and *θ*_CW_ = −45.8 K, suggesting a stronger Curie–Weiss paramagnetic component due to the higher absolute *θ*_CW_ value and lower *χ*_0_. A possible conclusion from this fit is a lower number of carriers in CuTTFtt, leading to a smaller Pauli contribution, alongside antiferromagnetic coupling between either copper- or TTFtt-based spins. The interplay of antiferromagnetic coupling with carrier density or mobility remains an interesting arc of investigation in these materials.

For Cu_2_TTFtt (Fig. 5C), the magnetic susceptibility (*χ*) is negative and extremely small (∼10^−4^ emu mol^−1^) down to 30 K even after accounting for diamagnetic corrections. The *χT* value is also negative at room temperature (−0.07 emu K mol^−1^, Fig. S25A). The magnetization at 1.8 K and 7*T* is only 0.01*μ*_B_, indicating that Cu_2_TTFtt exhibits diamagnetic behavior despite the presence of Cu^2+^ cations (Fig. S25B). The diamagnetic behavior of Cu_2_TTFtt suggests significant antiferromagnetic coupling leading to a strongly insulated singlet ground state.

To further investigate the nature of the spin centers in both compounds, we carried out X-band EPR measurements at 4 K (Fig. S27). The data were fitted using the EasySpin software^57^ with one and two *S* = 1/2 spin centers in Cu_2_TTFtt and CuTTFtt, respectively (Table S5, see SI for details). For Cu_2_TTFtt, only a single *S* = 1/2 resonance was observed at *g* = 2.011. This *g*-value and the observed sharp linewidth are characteristic of an organic radical and we therefore assign this signal to radical TTFtt^3−^ linkers. SQUID susceptibility measurements show that Cu_2_TTFtt is diamagnetic between 100 and 300 K, but exhibits a Curie tail at low temperature, supporting a small amount of magnetic impurities. Taken together, these results suggest strong antiferromagnetic exchange between Cu^2+^ and TTFtt^3−^ in Cu_2_TTFtt, which suppresses the Cu^2+^ EPR signal while leaving a small but detectable fraction of unpaired TTFtt^3−^ spins, likely from defects or disorder. This finding also provides further support for a formal redox-state assignment of Cu_2_TTFtt as (Cu^2+^)(Cu^+^)(TTFtt^3−^).

In contrast, the EPR spectrum of CuTTFtt displays two distinct *S* = 1/2 resonances. A narrow and weak signal at *g* = 2.005 (∼1% of total intensity) can be attributed to trace radicals, likely from TTFtt^3−^ defects. The dominant broader resonance at *g* = 2.036 (∼99% of the signal intensity) can be reasonably assigned to uncoupled Cu^2+^ spin centers based on comparison with other Cu dithiolenes.^58^ This assignment is also consistent with the SQUID susceptibility results, which indicate antiferromagnetic interactions between *S* = 1/2 Cu^2+^ ions.

Thus, the EPR results reveal that while CuTTFtt contains some Cu^2+^ spins with only a negligible amount of radical impurities, Cu_2_TTFtt exhibits suppressed Cu^2+^ signals due to strong Cu^2+^–TTFtt^3−^ antiferromagnetic interactions and some residual radical signatures from the TTFtt^3−^ linkers. These results provide additional confirmation of our proposed redox state assignments for both compounds.

### Theoretical calculations

Density functional theory (DFT) calculations were performed to gain a deeper understanding of the electronic transport and magnetic behaviors of CuTTFtt and Cu_2_TTFtt and to compare them with NiTTFtt. The Vienna *Ab initio* Simulation Package (VASP)^59–61^ was used to perform first-principles calculations. The Perdew–Burke–Ernzerhof (PBE)^62^ exchange correlation functional was used within the Generalized Gradient Approximation (GGA). The Projector Augmented-Wave (PAW)^63,64^ method was used with the potentials PAW_PBE Cu for Cu, PAW_PBE C for C, PAW_PBE S for S and PAW_PBE Ni for Ni, respectively. The plane wave basis set was truncated at an energy cutoff of 800 eV for CuTTFtt and Cu_2_TTFtt, and 700 eV for NiTTFtt, yielding convergence of the total energy to 1 meV per atom. The Brillouin zone was sampled using a gamma-point centered 5 × 5 × 5 *k*-grid for all structural optimizations, a 7 × 7 × 7 *k*-grid for density of states (DOS) calculations, and a k-path determined from SeeK-path^65–67^ is used for band structure calculations. All calculations employed a SCF convergence criterion for the total energy of 10^−8^ eV with a Gaussian smearing width of 0.05 eV. For ionic relaxations, a force convergence criterion of 5 meV Å^−1^ was applied. van der Waals interactions were included *via* the DFT-D3 method with Becke–Johnson damping.^68^ All the band structure and DOS plots were generated using Sumo.^69^ The net atomic charge was calculated using Chargemol program by performing Density Derived Electrostatic and Chemical (DDEC6)^70–72^ atomic population analysis. The idealized infinite 2D sheet of Cu_2_TTFtt was used for simplicity, *in lieu* of the more complicated TMEDA capped structure. We also calculated a putative isolated 2D sheet of Cu_2_TTFtt.

First, the magnetic ground states of CuTTFtt, Cu_2_TTFtt, and isolated 2D sheets of Cu_2_TTFtt were investigated, all of which converge to a closed-shell ground state with zero magnetization. In Cu_2_TTFtt, the energy difference between the *S* = 1 and *S* = 0 states is significantly larger in the π-stacked system (0.337 eV) than in the isolated sheet (0.101 eV). This indicates that the π–π interactions in the stacked system make it increasingly favorable to pair electrons, promoting a closed-shell or antiferromagnetic coupling state. For CuTTFtt, the calculated energy gap between *S* = 1 and *S* = 0 is 0.203 eV, which is smaller than that of Cu_2_TTFtt. In the *S* = 1 excited state, the majority of the spin density is localized on the TTFtt linkers, with 0.35 electrons residing on the in-chain Cu atom and no significant spin density on the out-of-chain Cu. This localization likely promotes convergence to a closed-shell solution. Comparison with the vacuum-isolated sheet shows that π-stacking interactions do not significantly change the spin delocalization in the *S* = 1 state.

The diamagnetism observed in Cu_2_TTFtt aligns well with the calculated magnetic structure. The source of the paramagnetism observed in CuTTFtt is less clear. The smaller calculated energy gap between the closed-shell and *S* = 1 solutions suggests that a paramagnetic state is more reasonable in CuTTFtt. We also note the significantly lower high-temperature *χ*T in CuTTFtt than would be expected for an *S* = ½ paramagnet which is consistent with a comparatively large energy gap. However, it is difficult to rule out paramagnetic defect sites in this amorphous material. If some copper centers are structurally distorted they may behave as isolated paramagnets. In either case, computations support that paramagnetic behavior is more reasonable in CuTTFtt, but deeper explorations of the magnetism of TTFtt CPs with paramagnetic metal centers are still warranted.

**Fig. 6:**
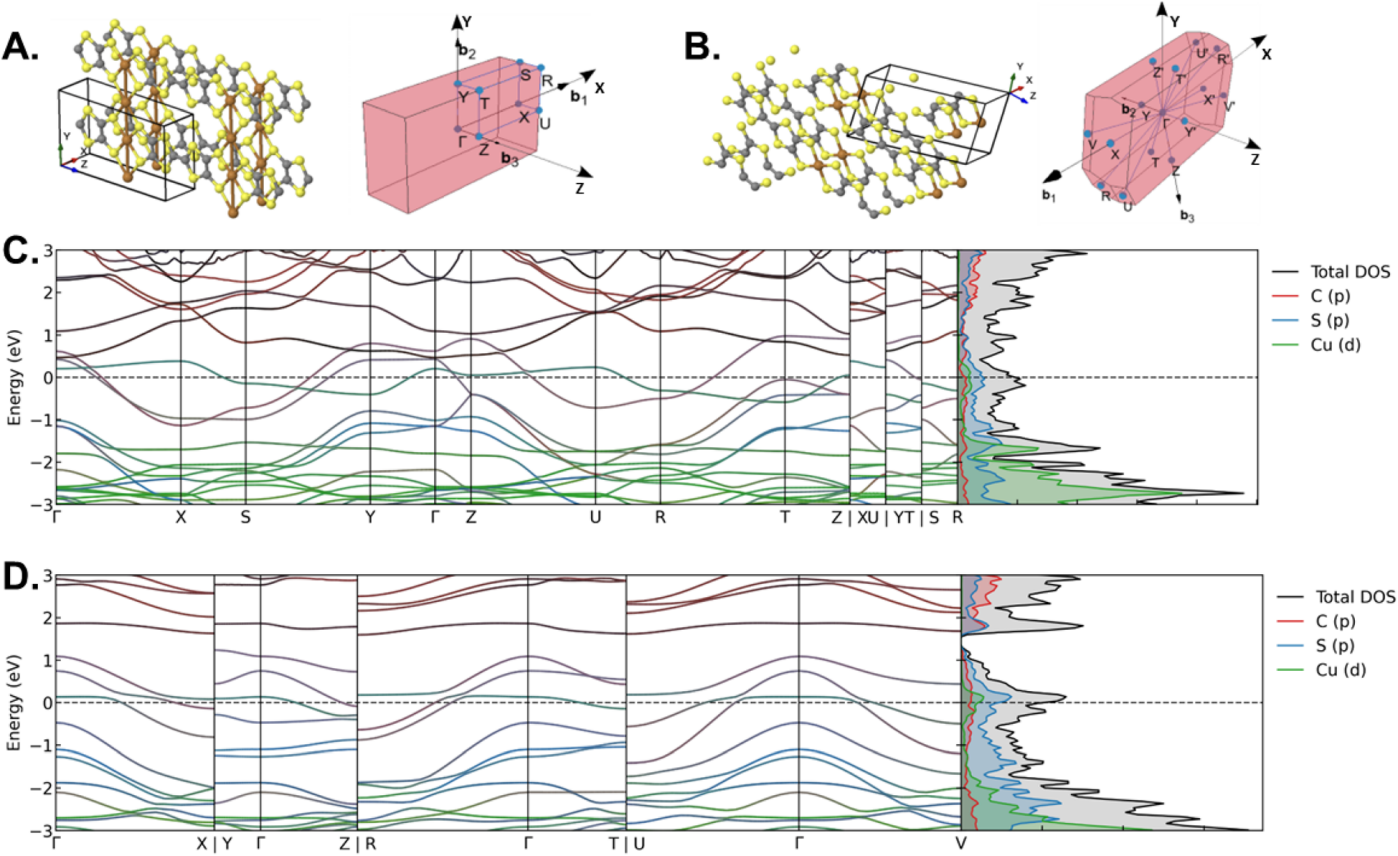
Band structure and DOS for Cu_2_TTFtt and CuTTFtt. (A) Primitive structure and Brillouin zone of the Cu_2_TTFtt unit cell. (B) Primitive structure and Brillouin zone of the CuTTFtt unit cell. (C) Projected band structure and DOS for Cu_2_TTFtt with the Fermi level denoted by black dashed lines. (D) Projected band structure and DOS for CuTTFtt with the Fermi level denoted by black dashed lines.

The band structure and DOS of Cu_2_TTFtt were also analyzed to assess conductivity along different crystallographic directions (Fig. 6C). Along the Γ–Z and Y–T directions (the TTFtt polymer chain directions, Fig. 6A), a steep TTFtt-based band crossing the Fermi level indicates metallic conductivity mediated by TTFtt. Along Γ–Z, a flatter Cu-based band above the Fermi level suggests electron localization on Cu. Along Γ–Y (the Cu chain direction, orthogonal to the TTFtt chains), a Cu-based band crossing the Fermi level implies metallic conductivity mediated by Cu atoms.

Along Γ–X and S–Y (the π-stacking direction), two steep TTFtt-based bands crossing the Fermi level indicate high inter-stack conductivity, while a flatter Cu-based band just above the Fermi level in Γ–X suggests possible Cu electron localization. The DOS at the Fermi level is dominated by S p-orbitals, with notable contributions from C p- and Cu d-orbitals, highlighting the role of TTFtt π-electrons and Cu-mediated interactions in charge transport. To examine the role of π-stacking, we also analyzed isolated 2D sheets of Cu_2_TTFtt (see SI Section 6). The band structure of the isolated sheets exhibits a 0.45 eV band gap located 0.3 eV above the Fermi level, with generally flatter bands. These findings confirm that π-stacking plays an important role in mediating conductivity along the polymer and Cu chains. However, we note the additional metallic directions between 1D chains in Cu_2_TTFtt, which may lead to the higher conductivity in this material.

To test the effect of TMEDA, we calculated the DOS for the 2D Cu_2_TTFtt system with TMEDA included (Fig. S28). The results confirm that TMEDA has negligible effect on the states near the Fermi level, supporting that its omission does not significantly affect the predicted electronic structure. Next, we consider the band structure and DOS of CuTTFtt to analyze the conductivity of the 1D chain along different crystallographic directions (Fig. 6D). The electronic band structure reveals multiple bands crossing the Fermi level, indicating metallic behavior. Along Γ–X (slightly off-axis to the π-stacking direction, Fig. 6B), two steep, TTFtt-based bands crossing the Fermi level suggest π-electron delocalization, while a flatter Cu-based band just above the Fermi level indicates electron localization on Cu. Along Γ–Z (the polymer chain direction), one Cu-based and one TTFtt-based band cross the Fermi level, indicating metallic character along the chain. Finally, along Γ–T (in-plane direction nearly perpendicular to the polymer chain), a less dispersive Cu-based band crossing the Fermi level suggests weaker interchain interactions. A 0.2 eV band gap appears at 1.4 eV above the Fermi level. The DOS at the Fermi level is primarily contributed by S p-orbitals, with notable C p- and Cu d-orbital contributions, again highlighting the influence of TTFtt π-electrons and Cu–TTFtt interactions in conductivity.

The band structure and DOS of NiTTFtt (Fig. S30A) are analyzed as a reference against CuTTFtt due to their different conductivities despite similar structural motifs and π-stacking interactions. Along the polymer chain and π-stacking directions, NiTTFtt exhibits metallic character, primarily driven by TTFtt with minimal Ni contributions. In contrast, a band gap appears along the in-plane direction nearly perpendicular to the polymer chain indicating hindered charge transport in this direction. CuTTFtt exhibits a higher DOS at the Fermi level, gradually decreasing to form a band gap, whereas NiTTFtt shows no such gap (Fig. S30B). Additionally, CuTTFtt shows more avoided crossings and flatter bands than NiTTFtt, suggesting more electron localization. The reduced band dispersion in CuTTFtt correlates with the experimentally observed higher conductivity in NiTTFtt relative to CuTTFtt.

Next, we compare the band structure and DOS of Cu_2_TTFtt and CuTTFtt to examine how the additional orthogonal Cu chains in Cu_2_TTFtt influence its metallic character. Along the polymer chain direction, Cu_2_TTFtt has a more dispersive TTFtt-based band crossing the Fermi level compared to CuTTFtt. Additionally, in this direction, Cu_2_TTFtt has a relatively flat Cu-based band above the Fermi level, while in CuTTFtt, the Cu-based band crosses the Fermi level. In CuTTFtt, along the in-plane direction nearly perpendicular to the polymer chain, a relatively flat Cu-based band again suggests some electron localization. In contrast, Cu_2_TTFtt shows a highly dispersed band along the Cu chain direction, indicating strong metallic character. The 0.2 eV band gap observed at 1.4 eV above the Fermi level in CuTTFtt is absent in Cu_2_TTFtt, as this gap is filled with a high density of bands from the additional Cu orbitals. Both materials display a similar total DOS at the Fermi level, but CuTTFtt shows a peak just above *E*_F_ that gradually decreases until forming the 0.2 eV gap (Fig. S30C).

## Discussion

The generally steeper band crossing the Fermi level in Cu_2_TTFtt compared to CuTTFtt strongly suggests higher electron mobility in Cu_2_TTFtt. Combined with the smaller activation energy of Cu_2_TTFtt relative to CuTTFtt, its electrical conductivity is expected to be higher, which is consistent with experiment. However, both compounds are theoretically predicted to be metallic, which contradicts the overall semiconducting behavior that is observed. We note that NiTTFtt, which is also predicted to be metallic, exhibits glassy metal behavior.^23^ This discrepancy suggests that reduced crystallinity in all of these samples likely introduces localized states or increased electron scattering, significantly altering their electronic transport properties.

The significantly lower conductivity of CuTTFtt compared to NiTTFtt is noteworthy. In several conductive reticular materials, Cu-based compounds typically exhibit higher conductivity than their Ni analogs with the same ligands, such as benzenehexathiolate and hexaiminobenzene.^2^ This divergent behavior in TTFtt^2−^-based materials may be attributed to the triplet diradical nature of TTFtt^2−^ (ref. 73) and suggests that magnetic metal centers, such as Cu^2+^, are detrimental to electrical conductivity when putatively magnetic linkers are present. This may plausibly arise from some degree of coupling between the paramagnetic centers and the TTFtt-based electrons which serve as carriers.

Although theoretical calculations suggest that the 2D structure of Cu_2_TTFtt should result in electronic conductivity comparable to that of amorphous NiTTFtt, experimental results reveal that the conductivity of Cu_2_TTFtt is actually one order of magnitude lower. This counterintuitive result can be primarily attributed to the presence of TMEDA, which coordinates with Cu^2+^ centers and acts as an insulating barrier at the grain boundaries, thereby impeding efficient charge transport between crystallites. In contrast, NiTTFtt contains no organic components beyond TTFtt itself, allowing for strong π–π interactions between chains that promote effective interchain electron transfer. Possible strategies to further enhance the conductivity of Cu_2_TTFtt include substituting TMEDA with smaller amines, performing post-synthetic ligand exchange, and optimizing growth/annealing conditions to enlarge crystallite sizes.

Multiple independent measurements indicate that Cu_2_TTFtt is mixed-valent, with coexisting Cu^+^ and Cu^2+^ centers. Mixed valency has been reported more frequently in iron-based conductive coordination polymers, where Fe^2+^/Fe^3+^ delocalization can enhance charge transport.^74^ By analogy, similar mechanisms may facilitate conductivity in Cu_2_TTFtt even though the macroscopic conductivity is likely limited by grain-boundary effects. The presence of Cu^+^ may also be structurally consequential: it likely contributes to stabilizing the 2D ribbon-like architecture observed for Cu_2_TTFtt. In particular, prior studies on poly[Cu_*x*_(Cu-ETT)] plausibly feature related structural/valence motifs,^36^ whereas analogous 2D arrangements have not been reported for Ni-based TTFtt or ETT systems—as expected due to the much lower stability of Ni^+^ relative to Cu^+^. Collectively, these considerations highlight the importance of Cu^+^ in stabilizing 2D networks and suggest that mixed valency may serve as an additional feature for tuning structure and charge transport in TTFtt-based coordination polymers.

Cu_2_TTFtt, with its 2D structure and strong antiferromagnetic (AFM) interactions, bears some similarity to the layered cuprate materials, which exhibit high-temperature superconductivity.^75^ In cuprates, doping introduces charge carriers that suppress long-range AFM ordering and enable unconventional superconducting states. We speculate that a similar approach in Cu_2_TTFtt, specifically doping at the Cu sites or modulating the redox state of the TTFtt ligand, may result in interesting electronic and magnetic properties. This is particularly compelling given the interplay of 2D geometry, AFM interactions, and the potential for doping-induced charge delocalization in Cu_2_TTFtt. Experimental exploration of doping strategies and their effects on the electronic density of states, along with theoretical studies to identify accessible pairing mechanisms, are exciting future areas of study.

## Conclusions

In this study, two new copper-TTFtt-based coordination polymers, CuTTFtt and Cu_2_TTFtt have been successfully synthesized and characterized. The isolation of these materials was made possible by leveraging pre-synthetic redox control of the TTFtt ligand, where differentially oxidized transmetalation precursors provide access to the two different materials. Structural analyses using PXRD, PDF and EXAFS methods reveal that CuTTFtt adopts an amorphous 1D chain structure, while Cu_2_TTFtt features a 2D ribbon-like layered framework due to the inclusion of ribbon-capping TMEDA molecules.

Comprehensive spectroscopic studies, including sulfur and copper K-edge XAS, Raman spectroscopy, and XPS, demonstrate that the oxidation states of the TTFtt ligand and Cu centers play a critical role in determining the electronic and magnetic properties of these materials. The material CuTTFtt features an oxidized TTFtt^2−^ state, while the spectroscopic evidence supports a reduced formally (Cu^2+^)(Cu^+^)(TTFtt^3−^) electronic structure in Cu_2_TTFtt. These results further underscore the importance of precise redox state determination in sulfur-based coordination systems.

Electrical conductivity measurements show that both materials are highly conductive, with room-temperature values of 23(2) S cm^−1^ for CuTTFtt and 50(2) S cm^−1^ for Cu_2_TTFtt. The higher conductivity of Cu_2_TTFtt is due to some combination of higher carrier densities or enhanced charge mobility as based on DFT calculations. Magnetic studies reveal contrasting behaviors. 1D CuTTFtt displays paramagnetic behavior, while Cu_2_TTFtt is diamagnetic, likely due to strong antiferromagnetic coupling interactions. These observations provide insight into the interplay between magnetic properties, dimensionality, and electronic properties in CPs containing redox-active ligands.

In conclusion, this work demonstrates the importance of redox state control and dimensionality in tuning the structural, electronic, and magnetic properties of TTFtt-based CPs, particularly as paramagnetic ions are included into these materials. The different structures arising from differentially oxidized precursors represents a new pathway for controlling material dimensionality and crystallinity. The inclusion of TMEDA in Cu_2_TTFtt also raises the possibility of modulating TTFtt-based materials with additional organic components. By bridging theoretical predictions and experimental realization, this study provides important insights into the rational design of highly conductive and magnetically tunable coordination materials.

## Author contributions

N. J. and J. S. A. conceived and designed the study. N. J. performed the majority of the experimental work. S. V. and J.-N. B. carried out the theoretical calculations. C.-Y. L. conducted variable-temperature electrical resistance measurements and scanning electron microscopy analyses. H. L. N. built the structural model. J. H. and K. W. C. performed structural characterization and analysis. J.-H. C. and S. P. conducted electrical conductivity and Seebeck coefficient measurements. A. R. and H. S. L. performed UV-vis and X-ray absorption spectroscopy measurements and subsequent data analysis. A. S. F. conducted X-ray photoelectron spectroscopy studies and contributed to structural analysis. All authors participated in writing and revising the manuscript.

## Conflicts of interest

There are no conflicts to declare.

## Supplementary Material

SC-OLF-D5SC03070F-s001

## Data Availability

Data for this article, including experimental details, composition characterizations, structural characterizations, spectroscopic characterizations, physical property measurements, and theoretical calculation details are available in SI. Supplementary information is available. See DOI: https://doi.org/10.1039/d5sc03070f.
