# Supplementary material for: Pre-synthetic redox control of structure and properties in copper TTFtt coordination polymers
Source: Chem Sci. 2025 Sep 11;16(41):19304–16. doi: 10.1039/d5sc03070f (PMC12447838; doi:10.1039/d5sc03070f)
Supplement: SC-016-D5SC03070F-s001 [file SC-016-D5SC03070F-s001.pdf]

Supplementary Information (SI) for Chemical Science.
This journal is © The Royal Society of Chemistry 2025

Dimensionality-Driven Physical Properties in Copper Tetrathiafulvalene-2,3,6,7-tetrathiolate Coordination Polymers

Ningxin Jiang,[1] Saranya Velliyarat,[2] Chen-Yu Lien,[1] Ha L. Nguyen,[1] Jan Hofmann[3], Jie-Hao Chen[4], Arun Ramanathan[5], Alexander S. Filatov[1], Henry S. La Pierre[5], Shrayesh Patel[4], Karena W. Chapman[3], Jan-Niklas Boyn[2], and John S. Anderson[1]

[1]Department of Chemistry, The University of Chicago, Chicago, Illinois 60637, United States

[2]Department of Chemistry, University of Minnesota, Minneapolis, Minnesota 55455, United States

[3]Department of Chemistry, Stony Brook University, Stony Brook, New York 11794, United States

[4]Pritzker School of Molecular Engineering, University of Chicago, Chicago, Illinois 60637, United States

[5]School of Chemistry and Biochemistry, Georgia Institute of Technology, Atlanta, Georgia 30332-0400, United States

Table of Contents

1. Experimental Section. 3

2. Composition Characterization 4

3. Structural characterization 6

4. Spectroscopic measurements 14

5. Physical property measurements 21

6. Theoretical calculations 25

7. References 27

1. Experimental Section.

General Methods. All manipulations were performed under dry N_2 using a MBraun UNIlab glovebox unless otherwise noted. Glassware was dried at 180 °C for overnight and cooled under vacuum prior to use. Tetrahydrofuran (THF) was initially dried and purged with N_2 on a solvent purification system from Pure Process Technology. Then THF was stirred over NaK alloy and passed through a plug of activated alumina prior to storing over 4 Å sieves under N_2. TTFtt($SnBu_2$)$_2$ and [Fc^{BzO}][BAr^F_4] (TTFtt = tetrathiafulvalene-2,3,6,7-tetrathiolate; Bu = n-butyl, Fc^{BzO} = benzoyl ferrocenium, BAr^F_4 = tetrakis(3,5-bis(trifluoromethyl)phenyl)borate) were prepared according to literature procedures.[1, 2] All other chemicals were purchased from commercial sources and used as received unless noted. Tetramethylethylenediamine(TMEDA) was purged with N_2 for 30 minutes before being transferred into the glovebox.

Cu_2TTFtt, TTFtt($SnBu_2$)$_2$ (0.075 mmol, 60 mg) in 3 mL THF was added into a 2 mL THF solution of copper(II) trifluoroacetylacetonate (0.15 mmol, 56 mg) with 0.3 mL of TMEDA. Black precipitate can be observed immediately, and the reaction mixture was heated at 70 °C overnight (~12 h). The precipitate was isolated via centrifugation and washed with THF (4 mL × 4). The product was then dried under vacuum at 70 °C for 6 hours and 32 mg of a dark green powder was collected as **Cu_2TTFtt** (94%). Detailed characterization and composition data are provided below.

Cu_2TTFtt-C, the as-prepared **Cu_2TTFtt** was dried under vacuum at 160 °C for 6 hours. The weight loss is 8.5% wt compared to the original **Cu_2TTFtt**.

CuTTFtt, TTFtt($SnBu_2$)$_2$ (0.075 mmol, 60 mg) in 3 ml DCM was mixed with $Fc^{BzO}BAr^F_4$ (0.15 mmol, 175 mg) in 3 ml DCM resulting in a homogenous dark purple solution. The resulting solution was added into a 5 ml MeOH solution of $CuCl_2$ (0.075 mmol, 10 mg) with vigorous stirring. The reaction mixture was stirred at 40 °C overnight (~12 h) over which time a solid formed. The solid product was isolated by centrifugation and was washed with DCM (4 ml × 2) and MeOH (4 ml × 2) sequentially. After being dried under vacuum at 70 °C for 2 hours, 26 mg of **CuTTFtt** was isolated as a black powder (88%). Detailed characterization and composition data are provided below.

2. Composition Characterization

X-ray Fluorescence (XRF). X-ray fluorescence measurements were performed on pressed pellets (7 mm in diameter) with a Rigaku NEX DE VS spectrometer under a He atmosphere.

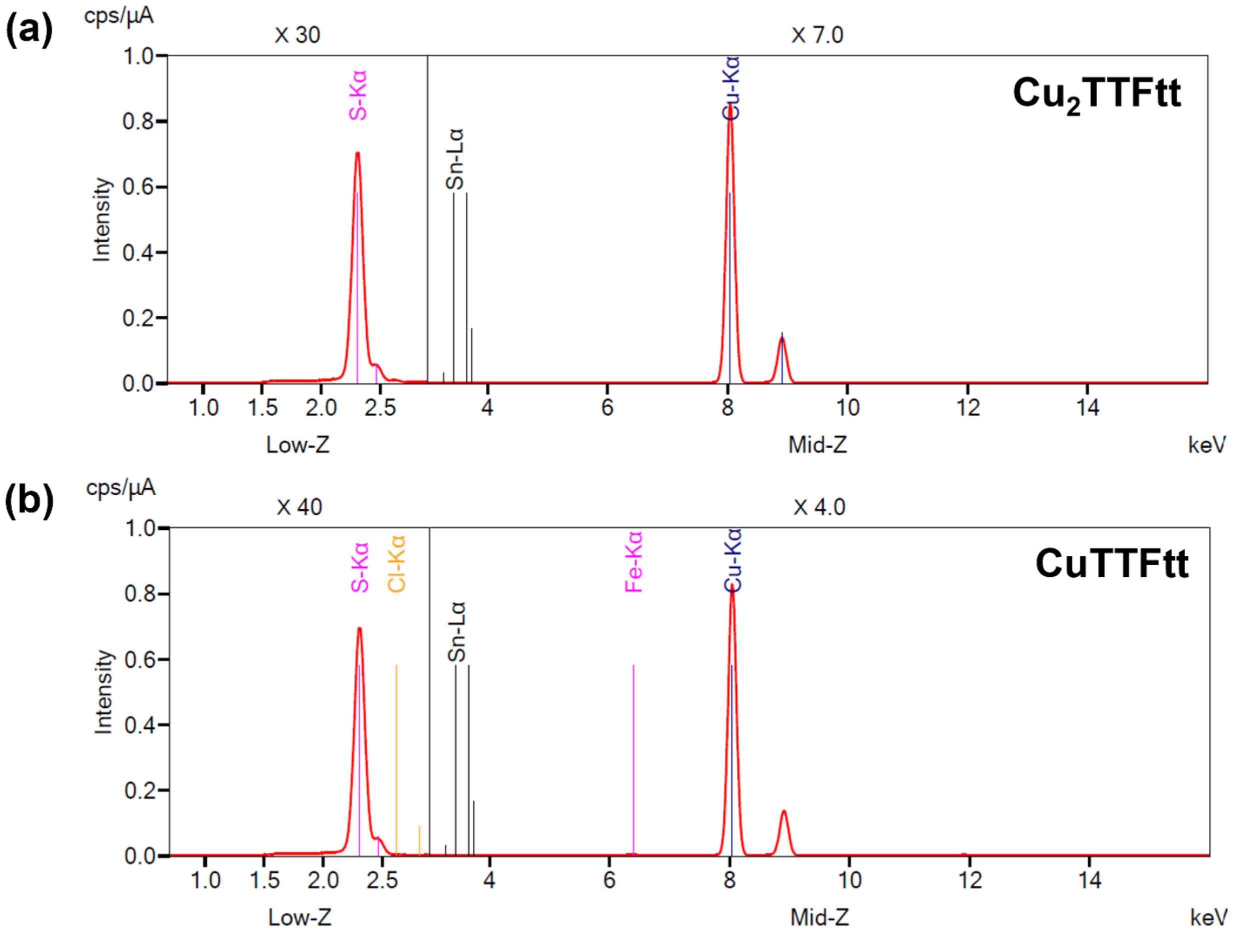

Figure S1. XRF spectra of (a) **Cu_2TTFtt** and (b) **CuTTFtt**.

Combustion elemental analysis. Combustion elemental analyses (C, H, N, S) were performed by Midwest Microlabs.

Table S1: Summary of XRF results

Elements	**Cu_2TTFtt** mol%	**CuTTFtt** mol%
S	79.0(1)	89.9(5)
Cu	20.9(1)	9.3(4)
Sn	<0.1	0.3(2)
Fe	N.A.	<0.1
Cl	N.A.	0.9(1)

Table S2: Summary of Combustion Elemental Analysis results.

Sample	C wt%	N wt%	H wt%
**Cu_2TTFtt**	21.36(1)	2.72(3)	1.75(3)
**Cu_2TTFtt-C**	20.35(9)	n.d.	1.14(9)
Theoretical ratio for $Cu_2C_6S_8$	15.81	0	0
Theoretical ratio for $Cu_2C_6S_8\cdot(C_6H_{16}N_2)_{0.5}$	21.04	2.73	1.57
**CuTTFtt**	19.99(8)	n.d	0.49(2)
Theoretical ratio for CuC_6S_8	18.38	0	0

Thermogravimetric analysis (TGA). Thermogravimetric analysis was performed using a TA Instruments Discovery analyzer. Approximately 5 to 10 mg of sample was loaded into a pre-tared Pt pan and measured under N_2. Samples were measured from 35 °C to 400 °C using a linear temperature ramp of 10 °C min^{-1}.

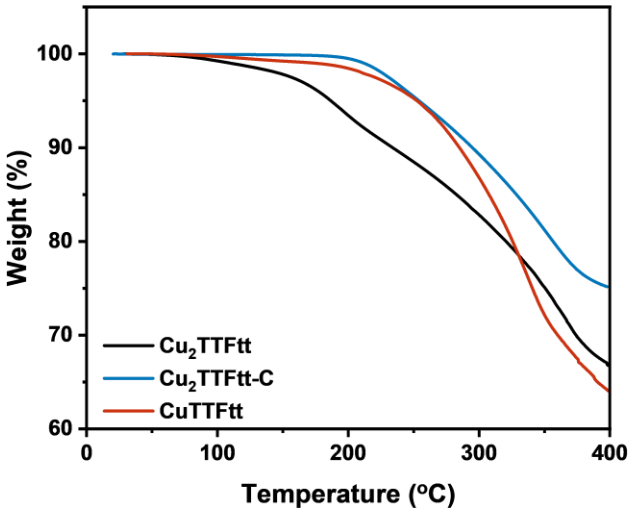

Figure S2. TGA results of Cu-TTFtt samples from 35 °C to 400 °C.

3. Structural characterization

Powder X-ray Diffraction (PXRD). Laboratory PXRD data were acquired on a Rigaku MiniFlex benchtop X-ray diffractometer equipped with Cu Kα radiation in a Bragg−Brentano reflection geometry. The data analysis was carried out by using GSAS-II software.

Scanning Electron Microscopy (SEM). SEM images were taken on a Carl Zeiss Merlin electron microscope using the In-Lens detector at the Materials Research Science and Engineering Center (MRSEC) at the University of Chicago. The sample powder was dispersed on conductive tape substrates, and an accelerating voltage of 5.00 kV was applied for the measurement.

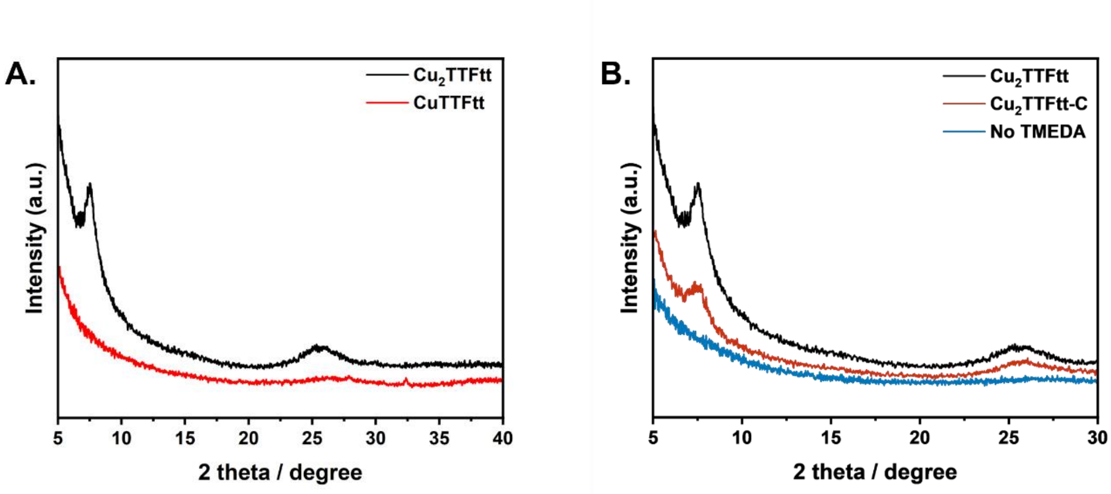

Figure S3. In-house PXRD patterns of (A) **Cu_2TTFtt** and **CuTTFtt** and (B) **Cu_2TTFtt** prepared and processed under varying conditions. In Fig S3B, black trace: standard preparation and work-up conditions. Red trace: sample dried at 160 °C instead of the standard 70 °C. Blue trace: sample synthesized without the addition of TMEDA.

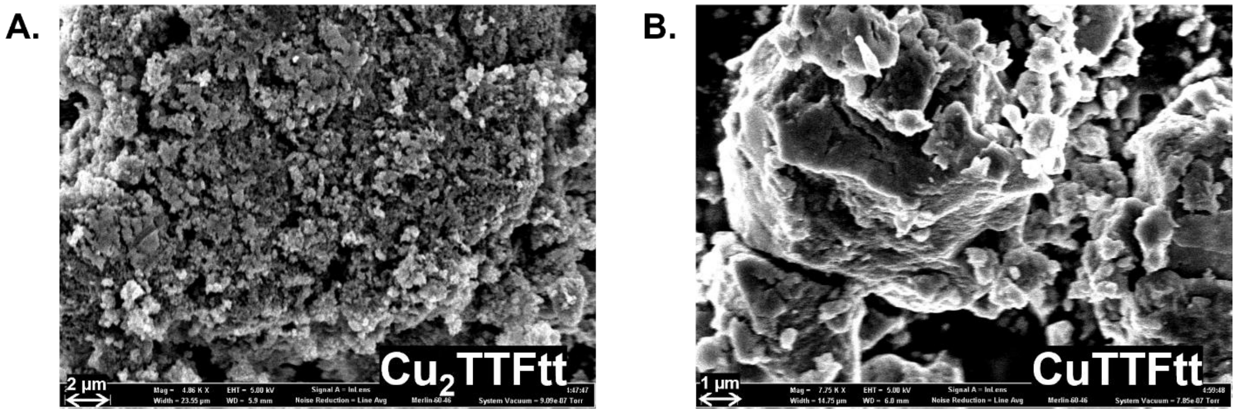

Figure S4. SEM images of (A) **Cu_2TTFtt** and (B) **CuTTFtt**.

Pair Distribution Function Analysis (PDF). Total scattering data suitable for Pair Distribution Function (PDF) analyses were collected at the 28-ID-1 beamline, at the National Synchrotron Light Source II, Brookhaven National Laboratory using high energy X-rays (λ = 0.1665 Å). A CeO_2 standard (NIST SRM 674b) was used to calibrate the detector geometry, and the diffraction images were reduced to one dimensional scattering intensity data within GSAS-II.[3] The PDF was obtained with in xPDFsuite[4] (Qmax ≈ 23.56 $Å^{-1}$) after subtracting the background contribution to the scattering data. Structure models were refined against the PDF within PDFgui.[5]

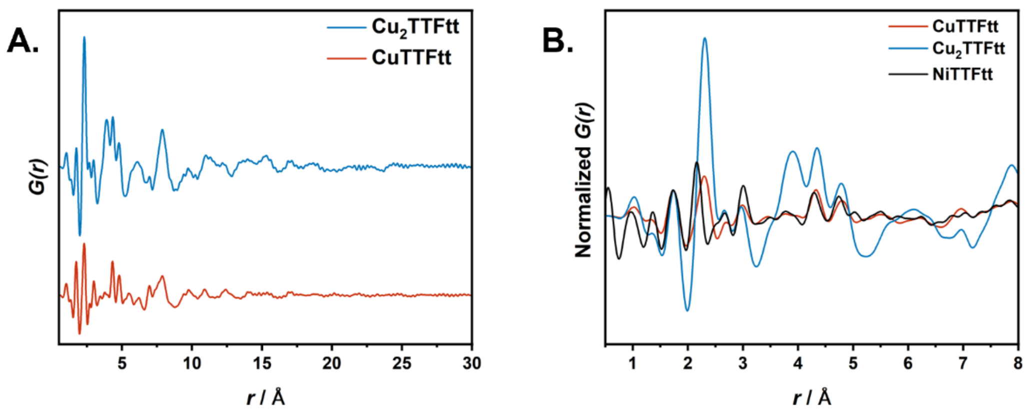

Figure S5. (A) PDF of **Cu_2TTFtt** and **CuTTFtt**. (B) Normalized PDF of **Cu_2TTFtt**, **CuTTFtt** and **NiTTFtt**. The curves were normalized to peak centered at 1.7 Å (peak assigned to the C-S bond in TTFtt).

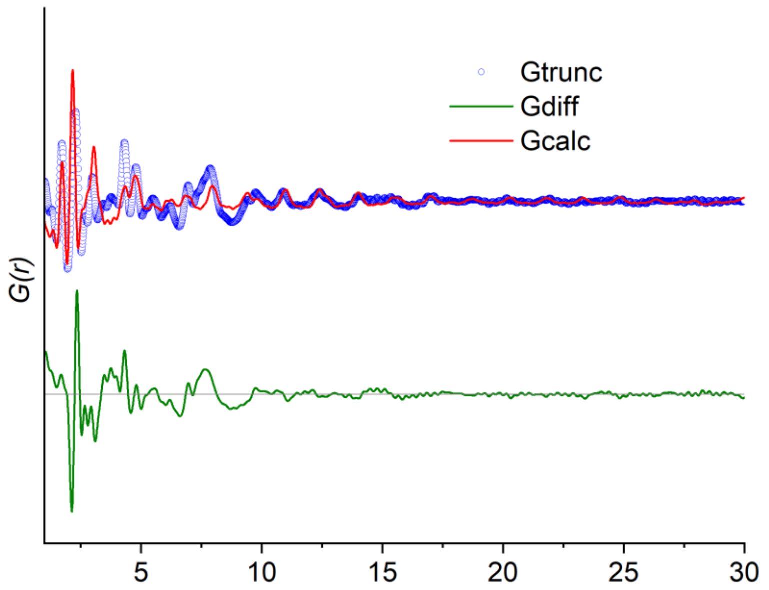

Figure S6. Refinement of single-chain **CuTTFtt** model, against PDF data.

Cu_2TTFtt was modeled using the Materials Studio software. The TTFtt linker, which contains four sulfur (S5) atoms, serves as a 4-coordinate unit. The square-planar copper (Cu1) atoms, each coordinating with four S5 atoms from four TTFtt linkers, generate a 2D square grid structure (**sql**). Additionally, the other copper atoms (Cu6) connect to four S5 atoms, forming $(–Cu–Cu–)_n$ and $(–Cu–TTFtt–Cu–)_n$ ribbon-like structures.

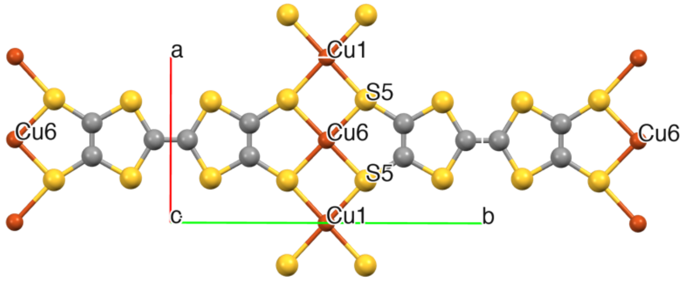

Figure S7. Model structure of **Cu_2TTFtt**, projected from *c* direction.

Based on this hypothesis, an initial unit cell was constructed, with the Cu1 and Cu6 atoms positioned at the center and edge of the unit cell, respectively. These Cu atoms were tethered by TTFtt linkers, resulting in the formation of a 2D structure. Following model construction, geometry optimization was performed using the Universal Force Field in the Forcite module of Materials Studio. The unit cell parameters were optimized, achieving convergence with an energy criterion set at 10^{-4} kcal mol^{-1}.

The model with TMEDA binding to Cu atoms at the structural edges' planes was built based on the above structure. First, a unit cell containing a 1×2×1 supercell of the

Cu_2TTFtt model was constructed. Two S atoms of the TTFtt linker at the edge of the unit cell were then removed, disconnecting the (–Cu–TTFtt–Cu–)$_n$ ribbon and leaving Cu7 atoms unsaturated. TMEDA molecules were subsequently added, binding to the Cu7 atoms. Finally, the structural model was geometrically optimized as described above.

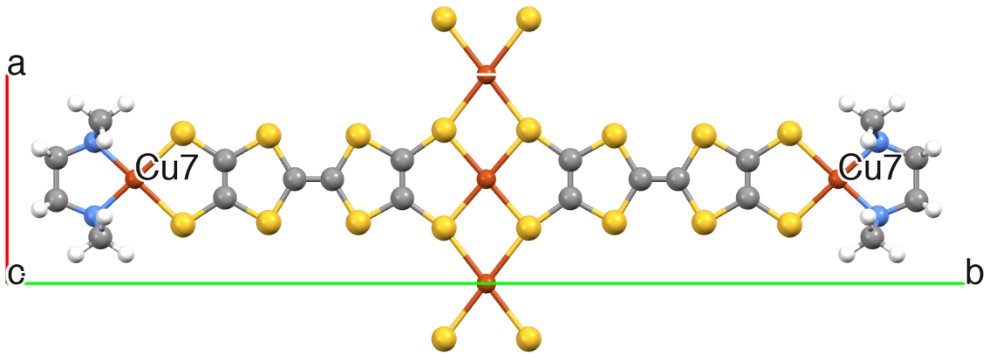

Figure S8. A single layer of the model structure of **Cu_2TTFtt** with TMEDA molecules binding to Cu atoms at the edges of the unit cell, projected from *c* direction.

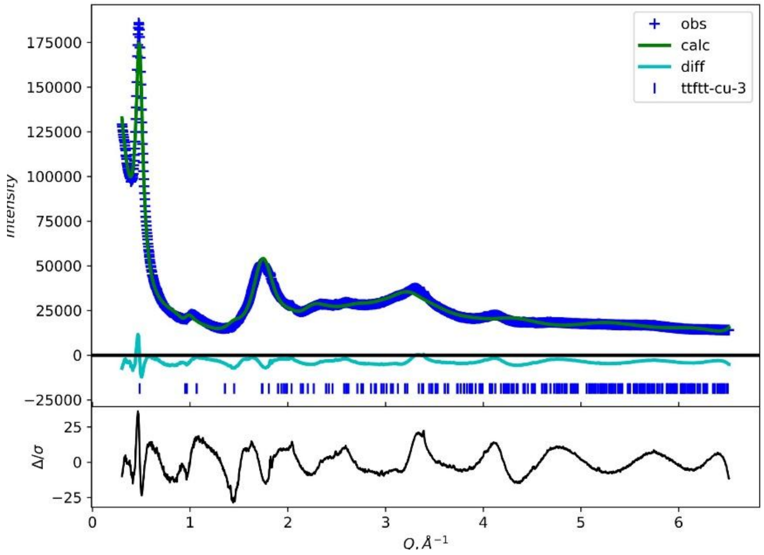

Figure S9. Rietveld refinement based on the structural model shown in Fig. 2C and 2D and Pmmm space group with a unit cell of *a* = 6.613(11) Å, *b* = 12.991(8) Å, *c* = 3.6136(20) Å (V = 310.4(6) Å^3) to the experimental **Cu_2TTFtt** PXRD pattern.

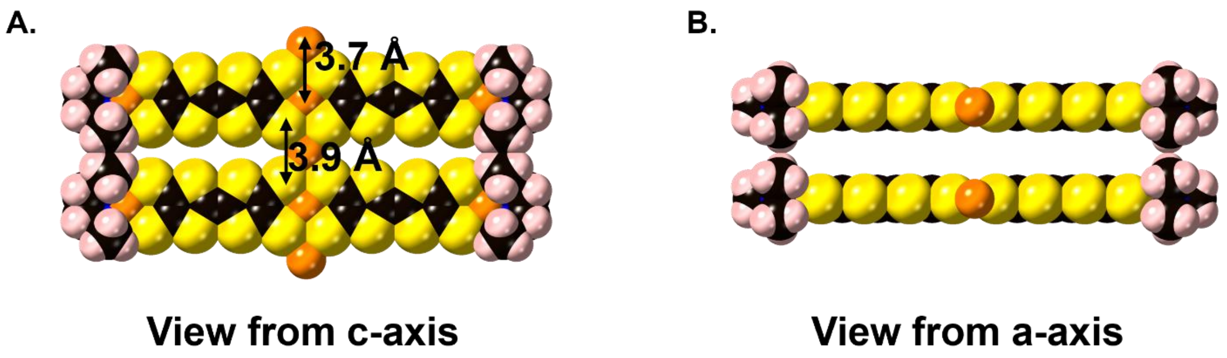

Figure S10. Structural model with TMEDA located on the terminal position for each Cu-TTFtt chains in **Cu_2TTFtt**. Van-der-Waal radii for each atom and space-filling model was chosen in CrystalMaker 11 for rendering figures.

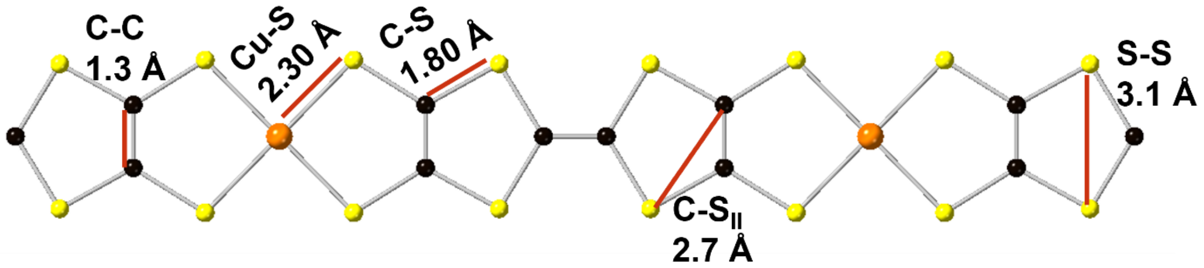

Figure S11. Atomic distances in the Cu-TTFtt chain in **Cu_2TTFtt** structural model.

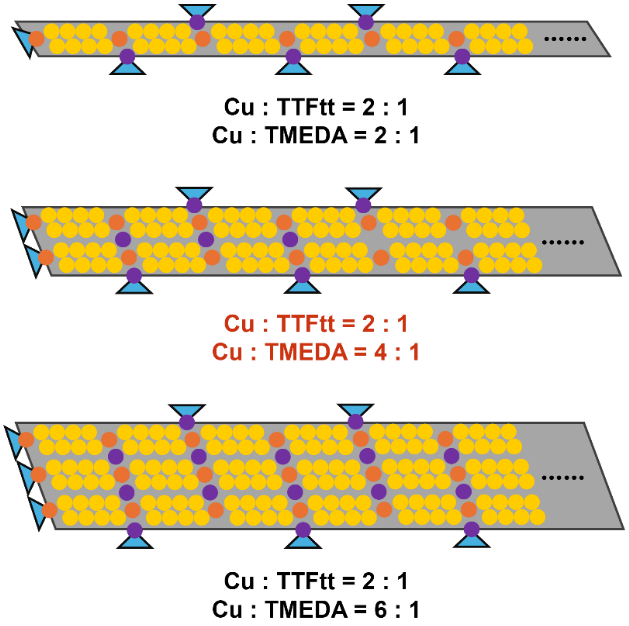

Figure S12. Demonstration of the impact of 2D ribbon size to TMEDA ratio in **Cu_2TTFtt** structural model.

Cu K-edge X-ray Absorption Spectroscopy (XAS).

Data were acquired at the Advanced Photon Source at the Argonne National Laboratory with a bending magnet source at the MRCAT 10-BM beam line. The incident, transmitted, and reference X-ray intensities were monitored using gas ionization chambers. A metallic Cu foil standard was used as a reference for energy calibration and was measured simultaneously with experimental samples. X-ray absorption spectra were collected at room temperature. Normalization and background subtraction of the data were performed using Athena from the Demeter software suite.[6] The EXAFS curve-fitting analysis program ARTEMIS was used to fit the EXAFS data [same reference]. The theoretical paths were generated using FEFF (version 6.0). During the fitting process, the bond distance (R) and the mean-square thermal and static deviation in R (σ^2) were allowed to vary for all pathways. The threshold energy (E_0) was allowed to vary but was constrained as a common value for all components in a given fit. The amplitude reduction factor (S_0^2) was fixed at 0.84 and the coordination numbers (N) were systematically varied to achieve the best fit. Fitting of **CuTTFtt** 1D structure was carried out using Cu-S and Cu-C single scattering pathways resulting with C-S and Cu-C distances to be 2.28 Å and 3.13 Å, respectively. The coordination number was refined to 4.0±0.2. For the refinement, this number was constrained to be the same for S and C atoms. To fit **Cu_2TTFtt** 2D structure, in addition to Cu-S and Cu-C scattering pathways, Cu-N and Cu-Cu single scattering pathways were incorporated. The distances for Cu-N, Cu-S, Cu-Cu and Cu-C are refined to be 1.76 Å, 2.27 Å, 2.95 Å and 3.14 Å, respectively. The copper coordination number refined to 4.1±0.4. Similar to **CuTTFtt**, this number was constrained to be the same for S and C atoms during refinement. The number of N atoms was not refined and fixed at 0.5 to accommodate variability of coordination modes (side and end chain). Refined copper coordination number of 4 leads to approximately 1:8 ratio of Cu-N to Cu-S bonds.

Table S3: EXAFS fit parameters for **Cu_2TTFtt**

**Cu_2TTFtt**	N	R(Å)	σ^2(Å)	R-factor	Reduced chi-square
Cu-N	0.5	1.76(1)	0.006(1)	0.006	1510
Cu-S	4.1(4)	2.27(1)	0.009(1)		
Cu-Cu	1	2.95(2)	0.010(5)		
Cu-C	4.1(4)	3.14(2)	0.006(4)		

Independent points: 12; Number of Variables: 10; ΔE_0= 5(1) eV; S_o^2 = 0.84; Fitting range: k: 3-14 Å^{-1}; R: 1.35-3.2 Å^{-1}. N, coordination number; R, interatomic distances; σ^2, Debye-Waller factors (the mean-square deviations in interatomic distance).

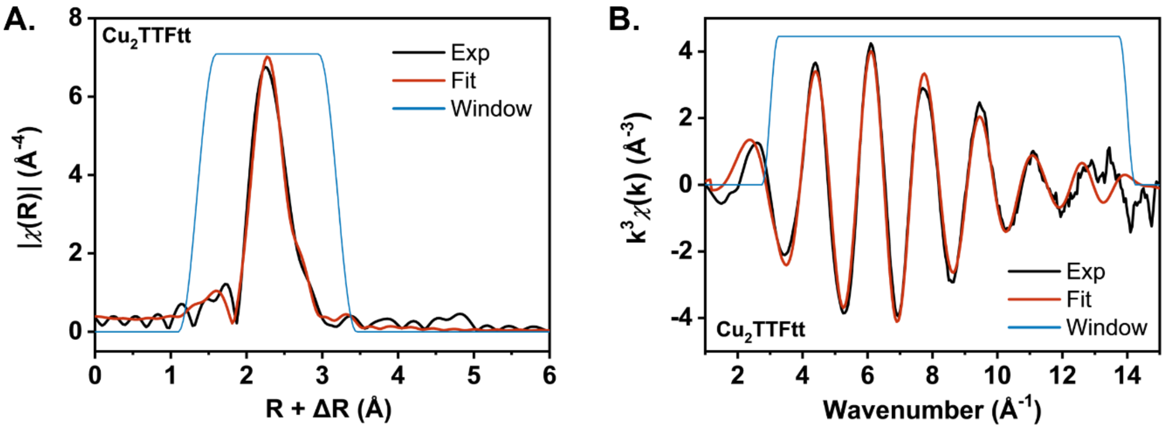

Figure S13. EXAFS spectra in (A) R-space and (B) k-space of Cu K-edge absorption of **Cu_2TTFtt**. The experimental data (black), simulated fit (red), and window (blue) are shown.

Table S4: EXAFS fit parameters for **CuTTFtt**

**CuTTFtt**	N	R(Å)	σ^2(Å)	R-factor	Reduced chi-square
Cu-S	4.0(2)	2.28(1)	0.006(1)	0.006	2036
Cu-C	4.0(2)	3.13(2)	0.011(5)		

Independent points: 12; Number of Variables: 6; ΔE_0=5.5(9) eV; S_o^2 = 0.84; Fitting range: k: 3-14 $Å^{-1}$; R: 1.3-3.1 $Å^{-1}$. N, coordination number; R, interatomic distances; σ^2, Debye-Waller factors (the mean-square deviations in interatomic distance).

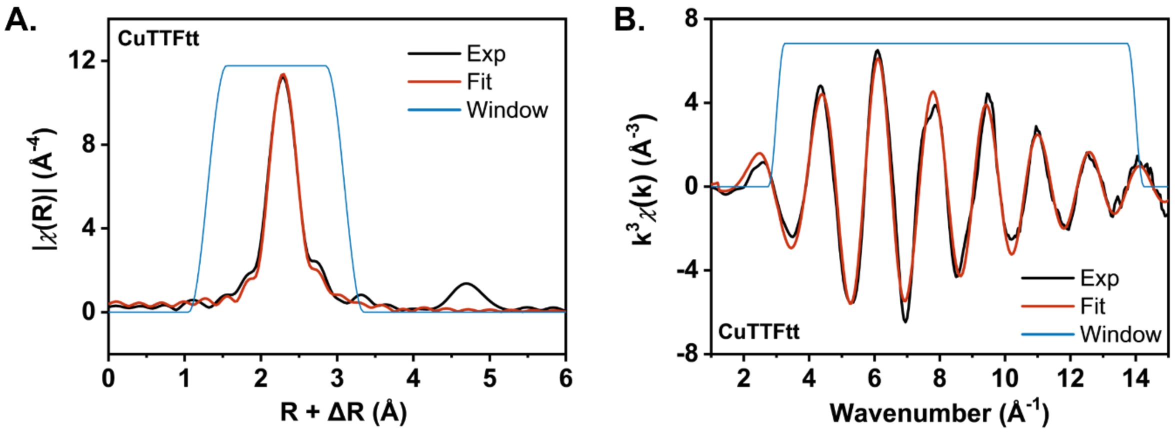

Figure S14. EXAFS spectra in (A) R-space and (B) k-space of Cu K-edge absorption of **CuTTFtt**. The experimental data (black), simulated fit (red), and window (blue) are shown.

4. Spectroscopic measurements

Infrared Spectroscopy. Infrared spectra were collected on a Bruker ALPHA II FTIR spectrometer with a Diamond Crystal ATR (Attenuated Total internal Reflectance) accessory. The sample was placed directly on the diamond crystal plate before measurements.

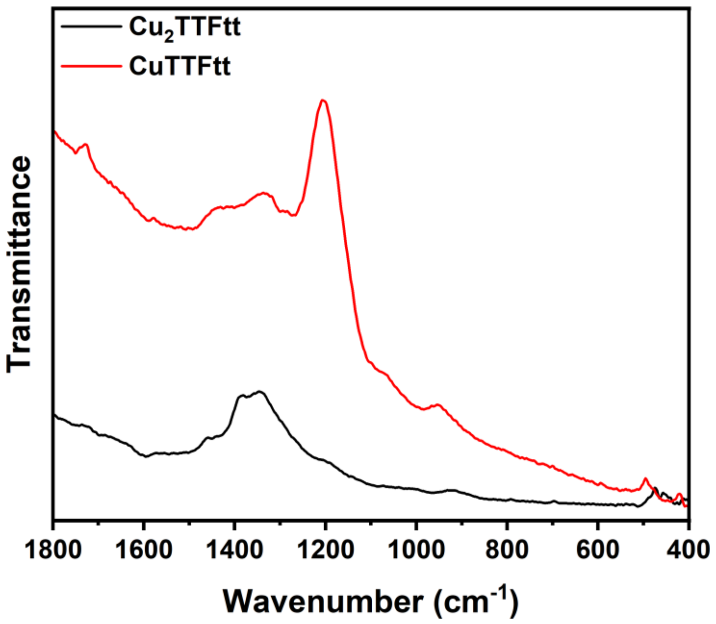

Figure S15. Infrared spectra of **Cu_2TTFtt** (black) and **CuTTFtt** (red).

Both samples exhibit high reflectivity to IR light in the probed region, which could be attributed to their high conductivity and delocalized electrons, hindering the investigation of vibrations in **Cu_2TTFtt** and **CuTTFtt**.

Raman Spectroscopy. Raman spectra were obtained with a Horiba LabRamHR Evolution confocal microscope. A Si (111) wafer was used for calibration. The **CuTTFtt** or **Cu_2TTFtt** powder sample, sandwiched between two glass slides secured with tape, was excited using a 532 nm light source at 5% power, a 100x long working distance objective, and a 600 mm^{-1} grating.

Sulfur K-edge XAS Sample Preparation. For both analytes, after drying under vacuum overnight, samples were ground with a mortar and pestle to yield a fine powder. An aluminum bracket with voids was used as sample holder with one side of the bracket

covered with double sided tape and the openings cut out and the other side covered with one sided sulfur-free tape. Inside a glovebox, the powder samples were painted as thinly as possible on the tape with a single-use paint brush. The backing of the double-sided tape was then removed, and the sample was covered with a single layer of 3525 Ultralene® (0.16 mil). The samples were shipped to the line in sealed Mylar bags under nitrogen.

Sulfur K-edge XAS measurements. Sulfur K-edge XAS data were measured at the Stanford Synchrotron Radiation Light source on the unfocused 20 pole, 2.0 T wiggler Beamline 4-3, under SPEAR3 storage ring parameters of 3 GeV and 500 mA. A Ni-coated, flat, bent premonochromator mirror was used for harmonic rejection and vertical collimation. A Si(111) double crystal monochromator was used for energy selection. A shutter was inserted automatically during each monochromator move to minimize photoreduction. The photon energy was calibrated to the maximum of the first pre-edge feature of $Na_2S_2O_3 \cdot 5H_2O$ at 2472.02 eV. At least three scans were measured for each sample to ensure reproducibility (and lack of beam degradation of the sample). Each sample was measured in duplicate.

X-ray Photoelectron Spectroscopy (XPS) and Ultraviolet photoelectron spectroscopy (UPS). XPS spectra were collected on an AXIS Nova spectrometer (Kratos Analytical) equipped with a monochromatic Al K_α X-ray source. The instrument work function was calibrated to give an Au $4f_{7/2}$ metallic gold binding energy of 83.95 eV. For calibration purposes, the binding energies were referenced to C 1s peak at 284.8 eV. Survey spectra were collected with a step size of 1 and 160 eV pass energy. The high-resolution spectra were collected with a pass energy of 40 and 0.1 eV step size. UPS spectra were collected with an AXIS Nova spectrometer using a UV-radiation source. The high-resolution spectra were collected with a pass energy of 20 and a 0.1 eV step size. Pressed pellets were affixed to conductive carbon tape under N_2 before loading into the spectrometer.

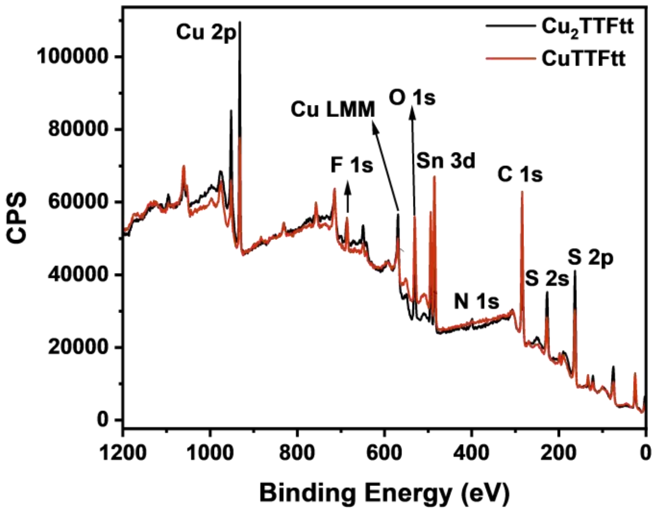

Figure S16. XPS survey spectra for **Cu_2TTFtt** and **CuTTFtt**.

The primary difference between the XPS survey spectra of **Cu_2TTFtt** and **CuTTFtt** is the presence of the N 1s peak in **Cu_2TTFtt**, which can be attributed to the TMEDA incorporated in this sample. In contrast, the N 1s peak is absent in **CuTTFtt**. The F 1s peak is present in both samples, likely originating from external fluoride contamination introduced during XPS sample preparation. Additionally, intense Sn 3d peaks are observed in both samples, indicating the accumulation of Sn near the surface. This surface enrichment of Sn differs from the bulk elemental compositions of the materials.

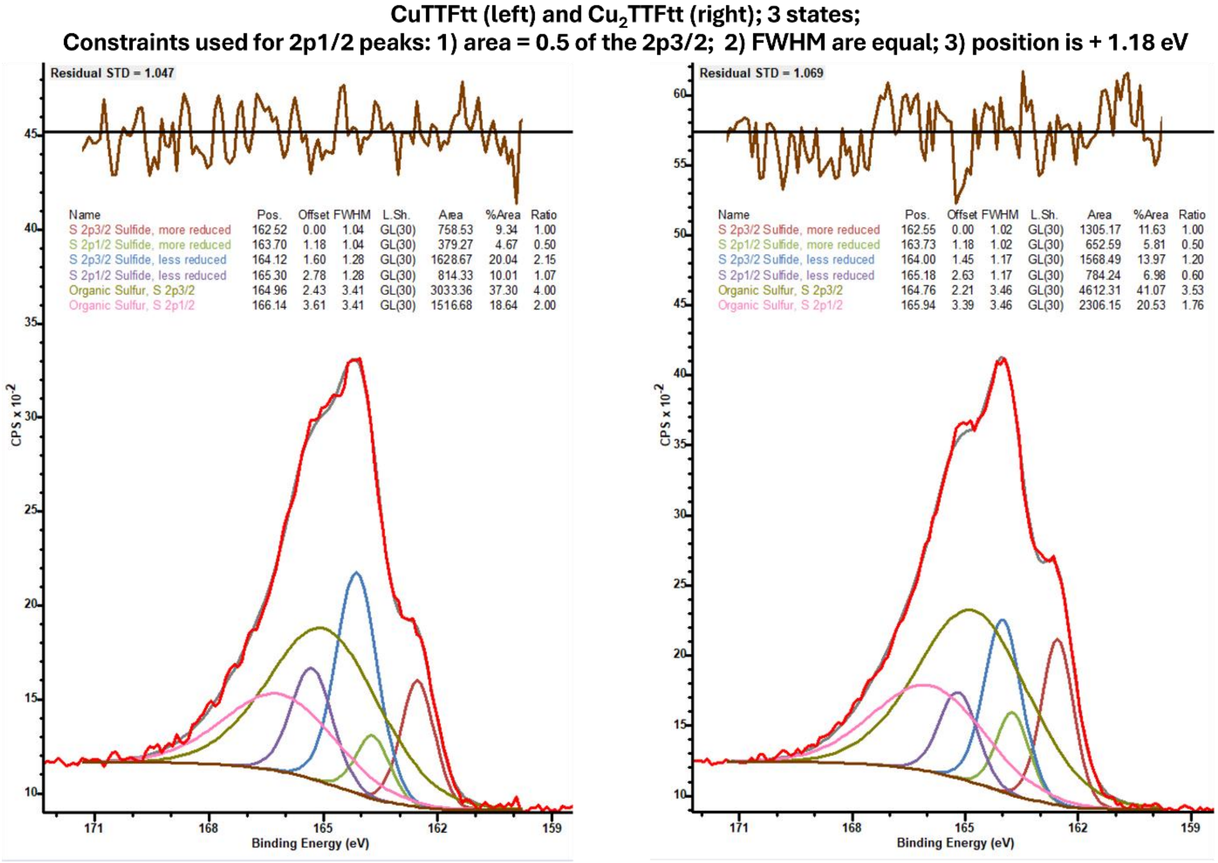

Figure S17. Peak fitting of high-resolution XPS S 2p spectra of (left) **CuTTFtt** and (right) **Cu_2TTFtt**. The constraint used in fitting process were shown on the top of the figure.

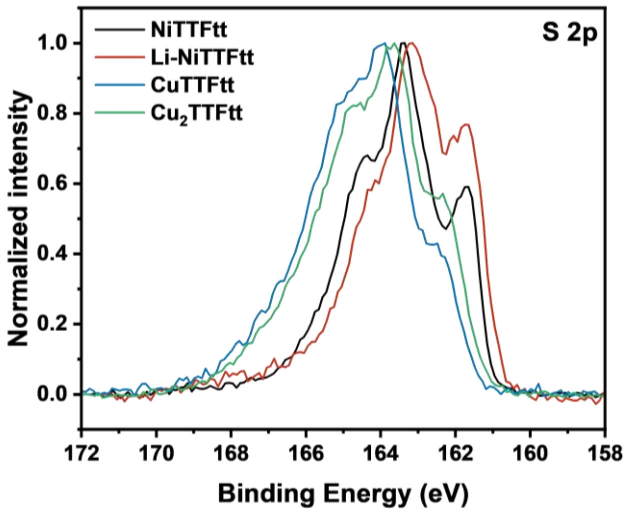

Figure S18. High-resolution XPS S 2p spectra for **NiTTFtt** (black), **Li-NiTTFtt** (red), **CuTTFtt** (blue), **Cu_2TTFtt** (green). The Shirley-type background was subtracted, and the spectra intensity was normalized.

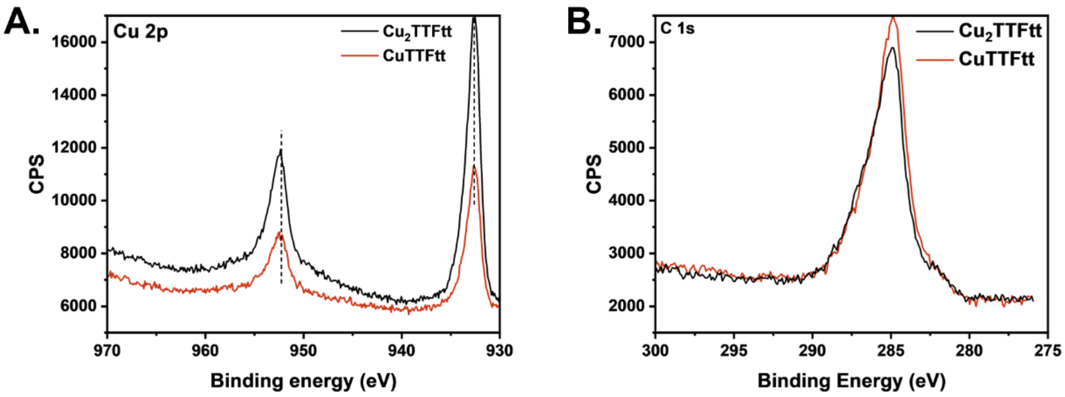

Figure S19. High-resolution XPS (A) Cu 2p and (B) C 1s spectra for **Cu_2TTFtt** (black) and **CuTTFtt** (red).

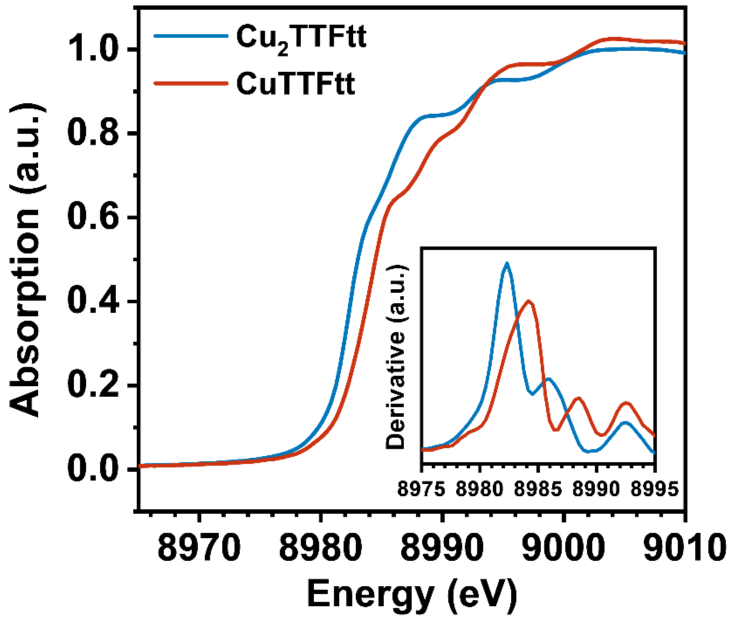

Figure S20. Cu K-edge XANES of **Cu_2TTFtt**(blue) and **CuTTFtt**(red).

UV-vis-NIR diffuse reflectance spectra. UV-vis-NIR diffuse reflectance spectra of samples were recorded on a UV-vis-NIR spectrophotometer (UH4150, Hitachi) with an integrating sphere attachment within the range of 200 – 2000 nm and $BaSO_4$ was used as the reflectance standard.

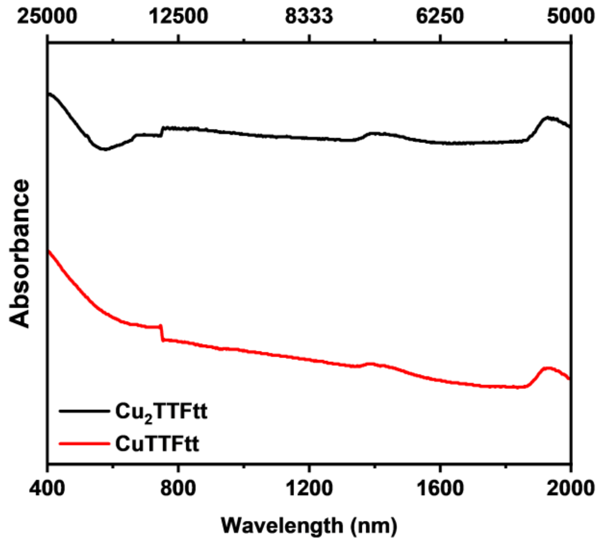

Figure S21. UV-vis-NIR spectra of **Cu_2TTFtt** and **CuTTFtt** from 400 cm^{-1} to 2000 cm^{-1}.

Discussion on Cu redox state assignment from XPS:

Cu 2p XPS spectra were collected to probe the Cu oxidation states in both **Cu$_2$TTFtt** and **CuTTFtt**. (Fig. S17). In both samples, two peaks are observed at 952.3 eV and 932.6 eV, corresponding to Cu 2p$_{1/2}$ and Cu 2p$_{3/2}$, respectively. These positions are similar between the two materials, and thus support a similar oxidation state assignment for copper in **CuTTFtt** and **Cu$_2$TTFtt**. We note that the positions of these features are most consistent with copper thiolate-based CPs, where copper is typically assigned as Cu(I).[7-11] However, assigning Cu(I) in these materials would imply that TTFtt in **CuTTFtt** is TTFtt^{1-} and in **Cu$_2$TTFtt** is TTFtt^{2-}. This contradicts the obtained spectroscopic data, as TTFtt^{1-} has not been previously observed, and features characteristic of TTFtt^{2-} are absent in **Cu$_2$TTFtt**.

Similar ambiguities in Cu oxidation state assignments have been reported for Cu-BHT materials (BHT = benzenehexathiolate), with conflicting reports assigning Cu(I) or Cu(II).[7, 9, 11] Additionally, in materials containing Cu–(S^{2-}) bonds, Cu 2p XPS spectra consistently show peaks at similar positions, which are often assigned to Cu(I).[12] Ambiguity for copper oxidation state assignment in these Cu-(S^{2-}) materials with X-ray-based techniques was also reported.[13] Considering these challenges, along with the wealth of other spectroscopic data we present here probing the oxidation states in **CuTTFtt** and **Cu$_2$TTFtt**, we posit that Cu 2p XPS is not a reliable tool for accurately determining Cu oxidation states in copper-sulfur materials.

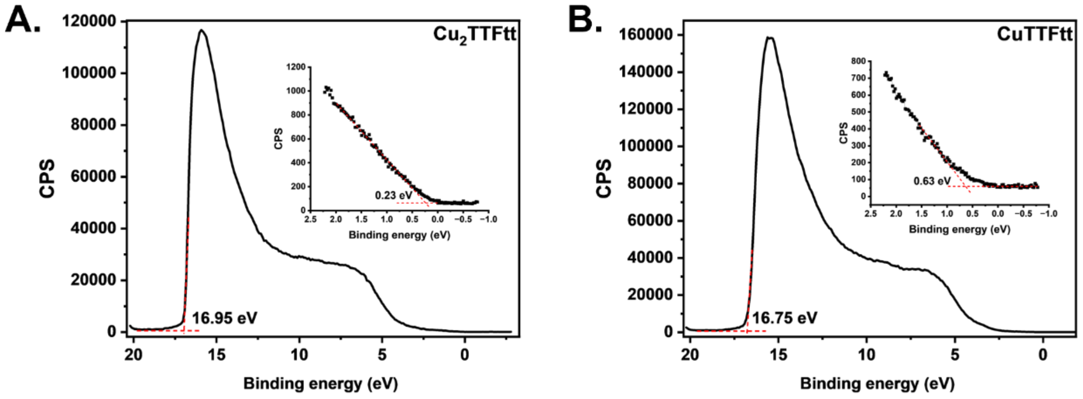

Figure S22. UPS spectra of **Cu$_2$TTFtt** and **CuTTFtt**.

5. Physical property measurements

Room-temperature electrical conductivity and Seebeck measurements. Room-temperature electrical conductivity and Seebeck measurements were performed with a custom-designed probe station in an argon-filled glovebox based on a previously reported setup.[14] Samples were pressed into circular pellets (diameter: 8 mm, thickness: 200–400 μm), and gold electrical contacts (~100 nm thick) were deposited by thermal evaporation through home-made shadow masks. The Seebeck coefficient was calculated from a linear fit of ΔV/ΔT. Forward and reverse scans were both carried out to obtain an average Seebeck value. The resistance (*R*) of the sample pellet was extracted from the linear fit of the I–V curve. Then the conductivity (σ) was calculated using the following equation:

$$\sigma = \frac{\ln\left(\frac{\sinh\frac{t}{S}}{\sinh\frac{t}{2S}}\right)}{\pi R t}$$

where t is the thickness of the pellet, and S is the spacing between probes.

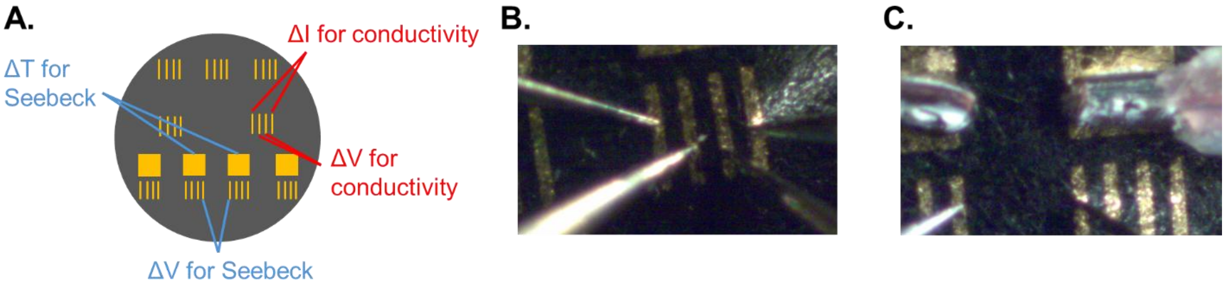

Figure S23. (A) A schematic illustration of the sample pellet deposited with gold contacts. Demonstration of probes placed on a sample pellet for (B) electrical conductivity and (C) Seebeck measurements.

Variable-temperature electrical resistance measurements. Variable-temperature resistance was measured using a physical property measurement system (PPMS, Quantum Design) which is connected to a Keithley 2636A source meter controlled by a LabVIEW interface. A strip of double-sided Kapton® tape was placed on a DC Resistivity/ETO Sample Puck (P102, Quantum Design) as an insulating layer, and the sample pellet was placed on the tape. The gold contacts on the pellet were manually connected to the puck using indium wires with a diameter of 0.25 mm.

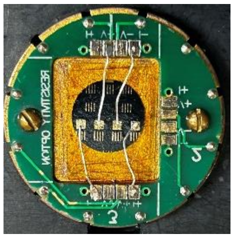

Figure S24. Demonstration of a sample pellet on a PPMS puck for variable resistance measurements.

Solid-state magnetic measurements. Solid-state magnetic measurements were performed on a Quantum Design MPMS3 SQUID magnetometer. The bulk powder of the sample (36.0 mg) was suspended in an eicosane matrix in a polycarbonate capsule to prevent movement. Diamagnetic corrections for the capsule and eicosane were made by measuring temperature versus moment in triplicate for each to determine a moment per gram correction. Diamagnetic corrections for the sample itself were applied using Pascal's constants of each atom on the basis of the formula of CuC_6S_8 or $Cu_2C_6S_8$.[15]

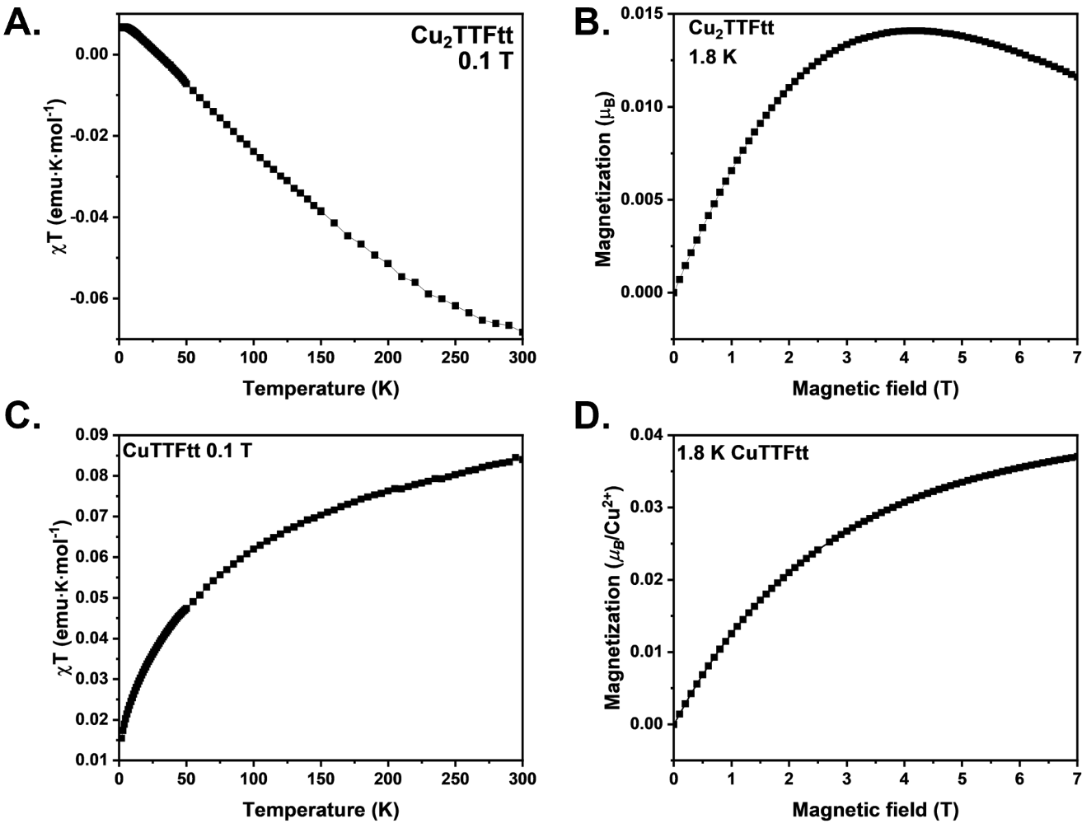

Figure S25. Magnetic property studies on **Cu_2TTFtt** and **CuTTFtt**. Temperature-dependence of χT of (A) **Cu_2TTFtt** and (C) **CuTTFtt** under 0.1 T and magnetic field dependence of magnetization of (B) **Cu_2TTFtt** and (D) **CuTTFtt**.

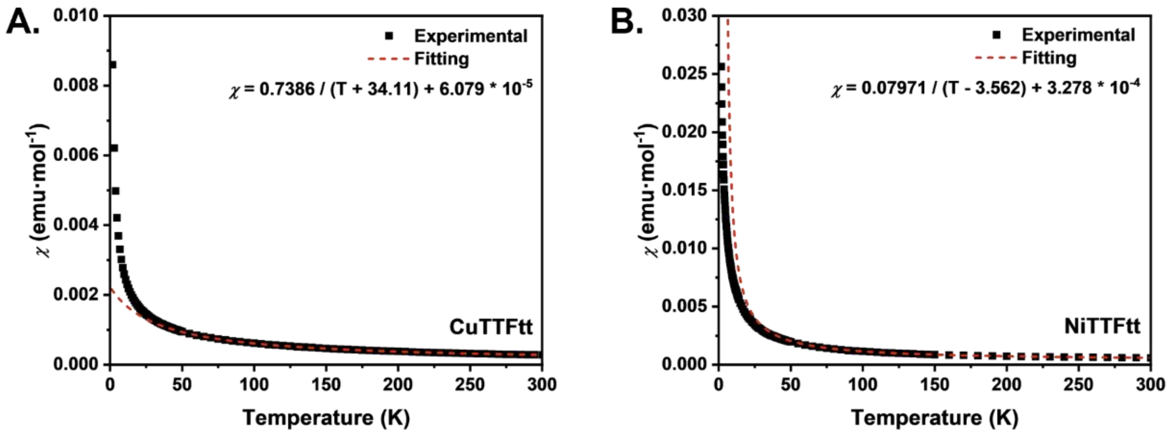

Figure S26. Temperature-dependence of χ of (A) **CuTTFtt** and (B) **NiTTFtt**. Dashed red curve represents the modified Curie-Weiss fitting to the experimental data (black dots) from 100 K to 300 K.

Electron paramagnetic resonance (EPR) measurements.

Continuous-wave (CW) EPR spectra were collected with a Bruker ELEXYS E580 spectrometer operating at X-band (9.37 GHz) frequencies with a Bruker super high Q cavity resonator (ER 4122SHQE) at 4 K. Non-saturating microwave power, 0.2 mW, and magnetic field modulation of 0.6 mT at 100 kHz were used. **Cu_2TTFtt** or **CuTTFtt** was mixed with boron nitride, ground to find powder with a mortar and pestle, and loaded into a sample tube. The Cu concentration in the resulting sample was ~1 wt%. The data were fitted using the EasySpin software with one and two S = 1/2 spin centers in **Cu_2TTFtt** and **CuTTFtt**, respectively.[16] Lorentzian line broadening and linear baseline correction were adopted.

Table S5. Fitted results of EPR spectra of **Cu_2TTFtt** and **CuTTFtt**.

	**Cu_2TTFtt**	**CuTTFtt**	
g-factor	2.0106(4)	2.00536(9)	2.0357(3)
Peak-to-peak line width (mT)	4.9(1)	2.30(2)	25.56(9)
Relative intensity	-	1.1	98.9

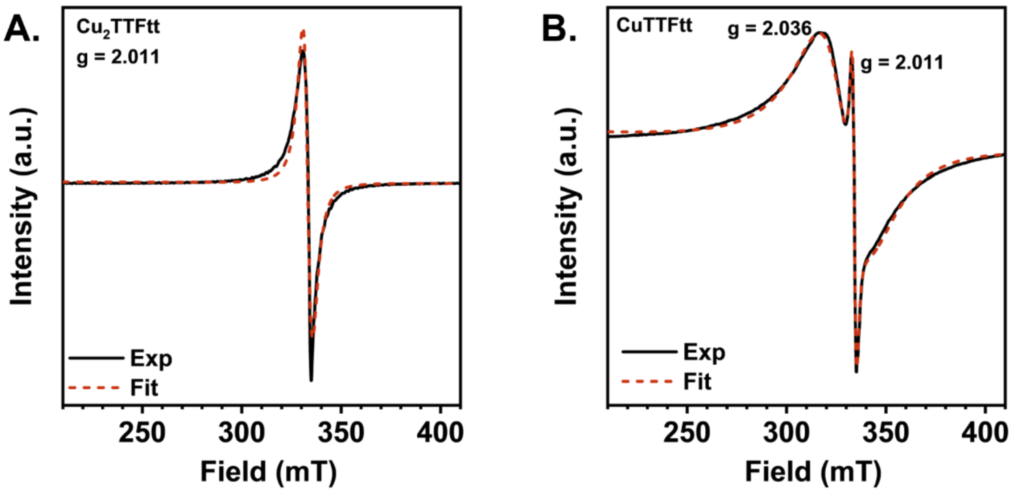

Figure S27. EPR spectra of (A) **Cu_2TTFtt** and (B) **CuTTFtt** at 4 K.

6. Theoretical calculations

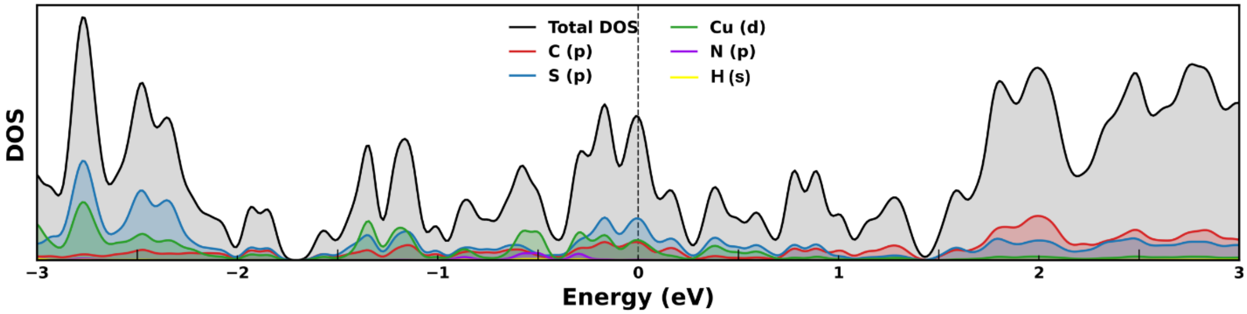

Figure S28. Projected DOS of the 2D **Cu_2TTFtt** structure including TMEDA. The Fermi level is indicated by the black dashed line.

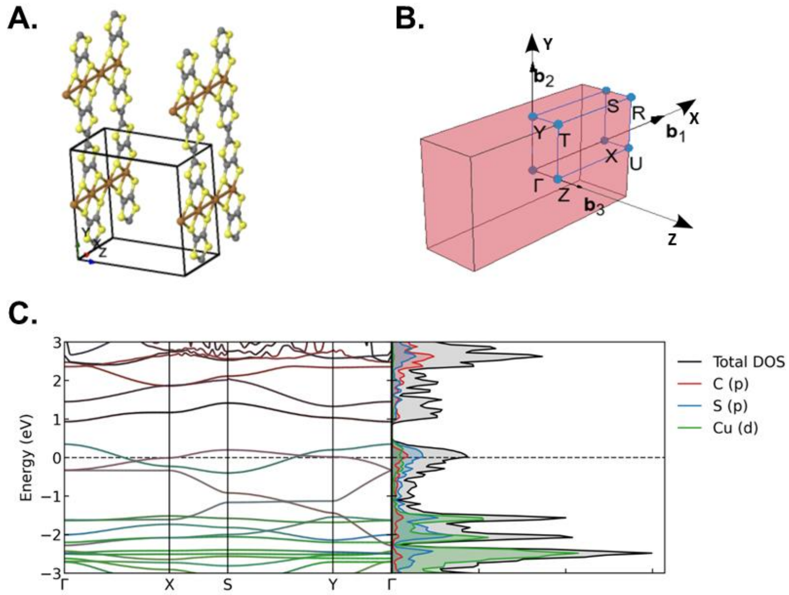

Figure S29. Band structure and DOS for 2D **Cu_2TTFtt** sheets isolated by vacuum. **A.** Primitive structure of the isolated 2D **Cu_2TTFtt** sheet unit cell. **B.** Brillouin zone. **C.** Projected band structure and DOS with the Fermi level denoted by black dashed lines.

To investigate the role of π-stacking in metallic conduction, the band structure and DOS of the **Cu_2TTFtt** vacuum-isolated sheet was analyzed. The band structure shows significant band splitting, particularly among the bands with TTFtt character. A TTFtt

based band splits into two branches: the higher-energy branch (LUMO) crosses the Fermi level, while the lower-energy branch (HOMO) remains below it.

In the Γ-X and S-Y directions (along the Cu chain), a Cu-based band crosses the Fermi level. Another TTFtt-based band splits into two branches: one approaches the Fermi level (LUMO), and the other remains lower in energy (HOMO). Along S-Y, two bands cross the Fermi level: one Cu-based and another TTFtt-based, suggesting that metallic conduction along the Cu chain is primarily driven by the Cu atoms, with additional contributions from TTFtt. In the Γ-Y direction (along the polymer chain), a TTFtt-based band crosses the Fermi level, indicating potential conductivity through the π-electrons. A relatively flat Cu-based band above the Fermi level creates a band gap, suggesting semiconducting character along the chain. The band structure shows a band gap of 0.45 eV at 0.3 eV above the Fermi level, with relatively flat bands suggesting limited metallic character. The DOS at the Fermi level is predominantly contributed by S p-orbitals, with notable contributions from C-p and Cu-d orbitals. This suggests that π-stacking plays a crucial role in mediating conductivity along polymer and Cu chains.

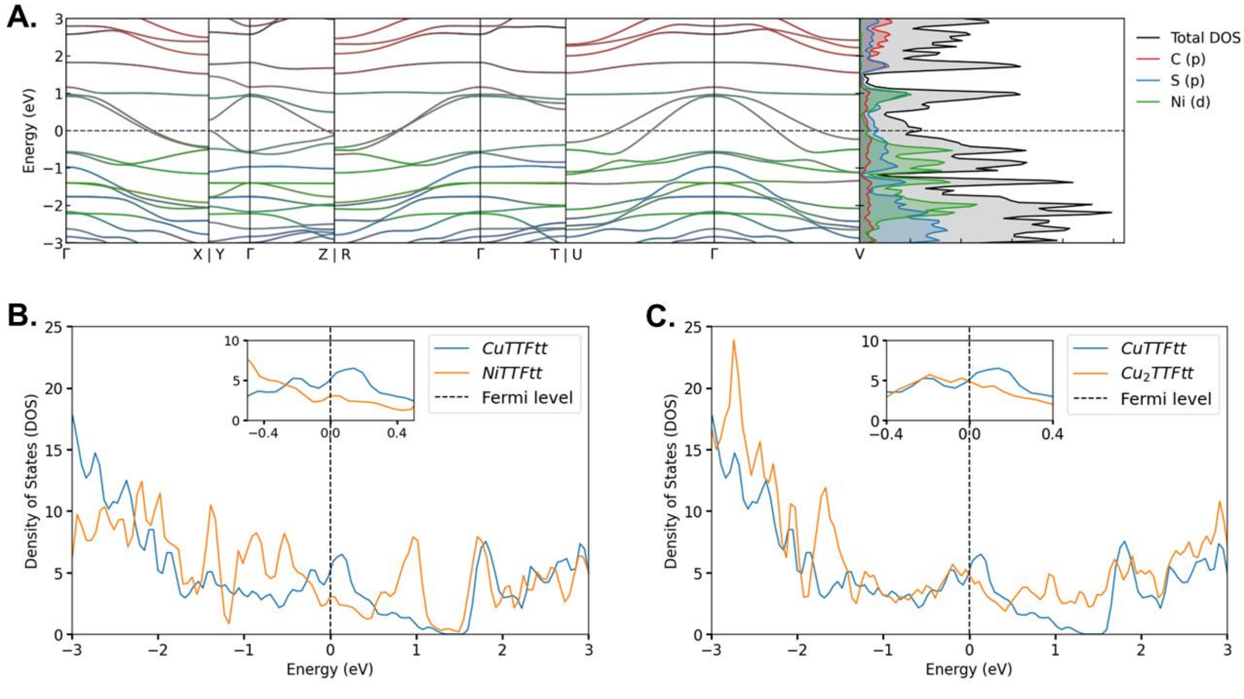

Figure S30. A. Band structure and DOS of **NiTTFtt**, with a primitive unit cell and Brillouin zone identical to those of **CuTTFtt**. B. Comparison of the DOS for **NiTTFtt** and **CuTTFtt**. C. DOS comparison between **CuTTFtt** and **Cu_2TTFtt**.

7. References

1. Xie, J.; Boyn, J.-N.; Filatov, A. S.; McNeece, A. J.; Mazziotti, D. A.; Anderson, J. S., Redox, transmetalation, and stacking properties of tetrathiafulvalene-2, 3, 6, 7-tetrathiolate bridged tin, nickel, and palladium compounds. *Chem. Sci.* **2020,** *11* (4), 1066-1078.
2. Xie, J.; Ewing, S.; Boyn, J.-N.; Filatov, A. S.; Cheng, B.; Ma, T.; Grocke, G. L.; Zhao, N.; Itani, R.; Sun, X., Intrinsic glassy-metallic transport in an amorphous coordination polymer. *Nature* **2022,** *611* (7936), 479-484.
3. Toby, B. H.; Von Dreele, R. B., GSAS-II: the genesis of a modern open-source all purpose crystallography software package. *J. Appl. Crystallogr.* **2013,** *46* (2), 544-549.
4. Yang, X.; Juhas, P.; Farrow, C. L.; Billinge, S. J., xPDFsuite: an end-to-end software solution for high throughput pair distribution function transformation, visualization and analysis. *arXiv preprint arXiv:1402.3163* **2014**.
5. Farrow, C.; Juhas, P.; Liu, J.; Bryndin, D.; Božin, E.; Bloch, J.; Proffen, T.; Billinge, S., PDFfit2 and PDFgui: computer programs for studying nanostructure in crystals. *J. Condens. Matter Phys.* **2007,** *19* (33), 335219.
6. Ravel, B.; Newville, M., ATHENA, ARTEMIS, HEPHAESTUS: data analysis for X-ray absorption spectroscopy using IFEFFIT. *J Synchrotron Radiat* **2005,** *12* (Pt 4), 537-41.
7. Huang, X.; Sheng, P.; Tu, Z.; Zhang, F.; Wang, J.; Geng, H.; Zou, Y.; Di, C.-a.; Yi, Y.; Sun, Y., A two-dimensional π–d conjugated coordination polymer with extremely high electrical conductivity and ambipolar transport behaviour. *Nat. Commun.* **2015,** *6* (1), 7408.
8. Huang, X.; Qiu, Y.; Wang, Y.; Liu, L.; Wu, X.; Liang, Y.; Cui, Y.; Sun, Y.; Zou, Y.; Zhu, J., Highly conducting organic–inorganic hybrid copper sulfides CuxC6S6 (x= 4 or 5.5): ligand-based oxidation-induced chemical and electronic structure modulation. *Angew. Chem.* **2020,** *132* (50), 22791-22798.
9. Huang, X.; Yao, H.; Cui, Y.; Hao, W.; Zhu, J.; Xu, W.; Zhu, D., Conductive copper benzenehexathiol coordination polymer as a hydrogen evolution catalyst. *ACS Appl. Mater. Interfaces* **2017,** *9* (46), 40752-40759.
10. Sheng, P.; Sun, Y.; Jiao, F.; Liu, C.; Xu, W.; Zhu, D., Optimization of the thermoelectric properties of poly [Cux (Cu-ethylenetetrathiolate)]. *Synth. Met.* **2014,** *188*, 111-115.
11. Mendecki, L.; Ko, M.; Zhang, X.; Meng, Z.; Mirica, K. A., Porous scaffolds for electrochemically controlled reversible capture and release of ethylene. *J. Am. Chem. Soc.* **2017,** *139* (48), 17229-17232.
12. Folmer, J.; Jellinek, F., The valence of copper in sulphides and selenides: An X-ray photoelectron spectroscopy study. *J. Less-Common Met.* **1980,** *76* (1-2), 153-162.
13. Kumar, P.; Nagarajan, R.; Sarangi, R., Quantitative X-ray absorption and emission spectroscopies: electronic structure elucidation of Cu_2S and CuS. *J. Mater. Chem. C* **2013,** *1* (13), 2448-2454.
14. Ma, T.; Dong, B. X.; Grocke, G. L.; Strzalka, J.; Patel, S. N., Leveraging sequential doping of semiconducting polymers to enable functionally graded materials for organic thermoelectrics. *Macromol.* **2020,** *53* (8), 2882-2892.
15. Bain, G. A.; Berry, J. F., Diamagnetic corrections and Pascal's constants. *J. Chem. Educ.* **2008,** *85* (4), 532.

16. Stoll, S.; Schweiger, A., EasySpin, a comprehensive software package for spectral simulation and analysis in EPR. *Journal of Magnetic Resonance* **2006,** *178* (1), 42-55.
